# Plasma metabolomics of early parenteral nutrition followed with enteral nutrition in pancreatic surgery patients

**DOI:** 10.1038/s41598-019-55440-z

**Published:** 2019-12-11

**Authors:** Zhengyu Jiang, Cen Wen, Changli Wang, Zhenzhen Zhao, Lulong Bo, Xiaojian Wan, Xiaoming Deng

**Affiliations:** Faculty of Anesthesiology, Changhai Hospital, Second Military Medical University/Naval Medical University, 200433 Shanghai, China

**Keywords:** Nutrition, Outcomes research

## Abstract

Nutrition support is essential for surgical patients. Patients undergoing pancreaticoduodenectomy (PD) require tremendous nutrient support but also faced with risks of infection and gastrointestinal complications. Early parenteral nutrition has recently shown benefits while limited information provided about the influence on metabolism. This prospective single-center cohort study used plasma metabolomics to clarify metabolic alteration after early parenteral nutrition followed with enteral nutrition. Patients undergoing pancreaticoduodenectomy (n = 52) were enrolled. 36 patients received parenteral nutrition within 3 days postoperatively followed with EN (TPN group), 16 patients received standard fluids followed with EN (GIK group). We found that the weight loss is reduced in TPN group while the other clinical outcomes and inflammatory cytokines showed no statistical significance. The TPN group showed significance in amino acids, lipid, and phospholipids metabolism compared with the GIK group. Moreover, integration analysis indicated that early TPN could promote the metabolism of long-chain fatty acids, phospholipids, ketone bodies, and branched-chain amino acids. We conclude that early TPN support followed with EN for patients undergoing PD reduced the perioperative weight loss and promoted the metabolic transition to anabolic metabolism with the recovery of lipid metabolism, suggesting its benefits for the recovery of patients.

## Introduction

Nutrition support is an essential component in routine postoperative surgical critical care of patients^1^. Enteral and parenteral nutrition, as two long-debated nutrition types, have gained much attention for their effects on patients’ recovery and long-term prognosis^2–4^. Enteral nutrition (EN) is effective in restoring malnutrition, gastrointestinal function, and shortening hospital stay^3,5,6^, while indigestion on the early stage of its initiation may limit the absorption of sufficient nutrients that needed for patients’ postoperative recovery. Total parenteral nutrition (TPN), on the other hand, provides more direct and sufficient nutrients, but increases the risk of postoperative infection or gastrointestinal dysfunction, if applied for a long time^7^. Therefore, though guidelines recommend the early enteral nutrition that enhances the recovery^8,9^, specific adjustment is still needed based on certain cases or surgeries.

Pancreaticoduodenectomy (PD) is a standard treatment for periampullary or pancreatic carcinomas^10,11^. Patients undergoing PD endured with malnutrition, endocrine, and exocrine dysfunction, both preoperatively and postoperatively^2^, and significant surgical stress further consumes the body nutrients storage. Nutrition support for pancreatic surgical patients remains challenging, as EN is usually not feasible in the early postoperative stage, and nutrients that provided are insufficient due to impaired gastrointestinal function. Therefore, additional parenteral nutrition is still helpful for postoperative recovery^6–9,12,13^. Treatment of TPN in the early postoperative period combined with EN showed some benefits to patients undergoing pancreatic surgery^13^. However, the question remains on how this combination benefits patients and by what mechanism that helped the metabolic recovery.

Plasma metabolome offered the measurement over time of metabolic responses of individuals or populations to certain interventions^14^. Except for its extensive use for the discovery of biomarkers sets^15^, the metabolomic profile offered both global and detailed information to analyze complicated relationships of host factors, disease, and treatment effects^16,17^. Previous studies in nutrition support focused on certain clinical outcomes of patients^1,4,18^ and overlooked specific therapeutic mechanisms and effects of nutrition support on patients’ postoperative metabolic recovery. The in-depth knowledge on metabolic transition may help to identify the critical component in nutrition support, or crucial signaling in metabolic adaptations, and thus improve the quality of nutrition support for critical patients.

To address these questions, the present study investigated the effects of 3-day postoperative TPN support versus standard fluids therapy, both then switched to EN support in the following times in patients undergoing PD. We concluded the patients’ clinical outcome, analyzed the metabonomic differences in postoperative day 4 (POD 4) and day 7 (POD 7), respectively, by a liquid chromatograph-mass spectrometer (LC-MS), and summarized the metabolic transitions as well as mechanistic indications of nutrition support between two groups.

## Methods

### Study design, subjects, and settings

Shanghai Changhai Hospital Ethics Committee approved this prospective single-center cohort (Approval No. CHEC2018-164), registered at the China Clinical Trials Registry (Registration No. ChiCTR1800015714) and independently carried out by Intensive Care Unit (ICU), Faculty of Anesthesiology, Changhai Hospital, Second Military Medical University (Shanghai, China). The protocol was following international ethical recommendations stated in the Declaration of Helsinki and International Conference on Harmonization, Good Clinical Practice. Patients provided written, informed consent before participation in the study. Patients (>18 years and <80 years, BMI 18.5–25 kg/m^2^) diagnosed with pancreatic carcinoma and underwent pancreaticoduodenectomy were included. Exclusion criteria are as follows: active bacterial infection or mycosis with systemic effects, inflammatory bowel disease, severe hepatic and renal dysfunction, gastrointestinal surgery history, allergy to TPN, previously received neoadjuvant chemotherapy or chemoradiotherapy; pregnant or breastfeeding; mental disorder; systemic administration of corticosteroids, unstable angina or myocardial infarction; unstable hypertension, severe respiratory disease requiring continuous oxygen treatment, disease of the blood system, systematic immune disease, refuse to join the study. Patients’ preoperative fasting were limited to 8~10 hours. Blood sample were collected at the morning of postoperative day 1 before the initiation of nutrition support (POD 1) and the morning of postoperative day 4 (POD 4) and day 7 (POD 7).

**Figure 1:**
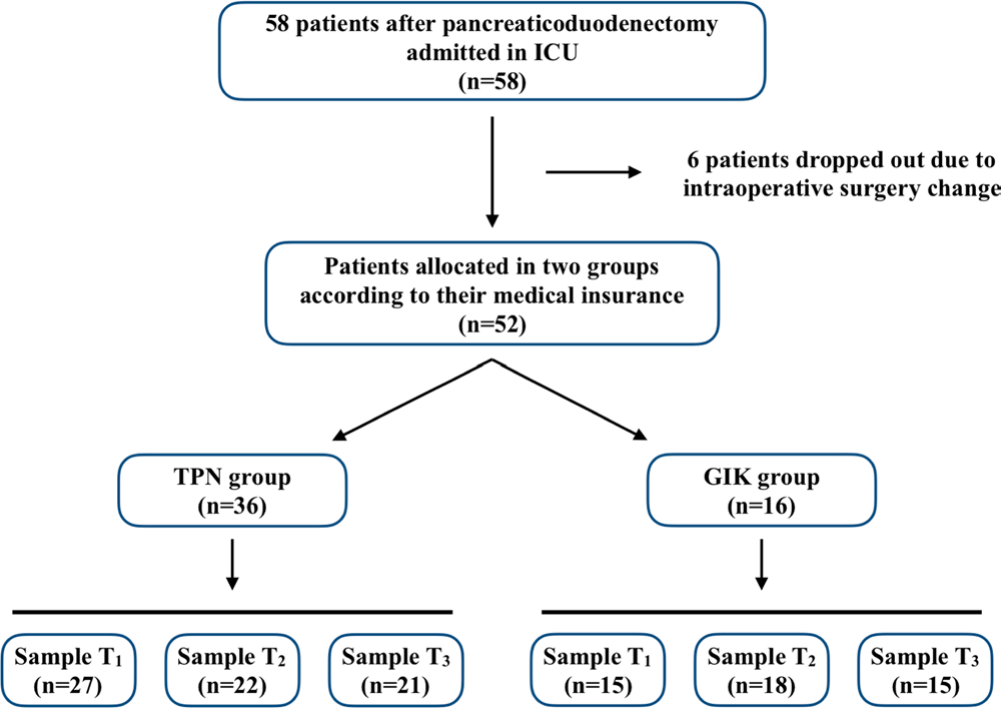
Flow chart and nutrition support protocol for TPN and GIK group. T_1_ = POD 1; T_2_ = POD 4; T_3_ = POD 7.

A total of 58 cases were included from March 2018 to October 2018. Six patients dropped out due to intraoperative change of surgery type (change to distal pancreatectomy or palliative operation). Thirty-six patients received 3-days postoperative TPN support (TPN group), while 16 patients received standard fluids with early EN support of the following days (GIK group) (Fig. 1).

### Allocation, treatment and nutrition support

Patients were admitted to the Intensive Care Unit after the surgery and divided into TPN or GIK group according to their medical insurance type. Nutrition support was initiated on the next morning (POD 1) after the admission. The same standard care and therapeutic schedule were prescribed to all included patients. TPN group receiving parenteral treatment mainly includes medium and long-chain fat emulsion, ω-3 fish oil fat emulsion, alanyl-glutamine, and other amino acids. GIK group received standard fluid therapy combined with glucose, sodium chloride and insulin. The specific component and calorie calculation of nutrition support are presented in Table 1. Postoperative enteral nutrition based on short peptide enteral nutrition powder was initiated according to the doctor’s judgment on patients’ status (e.g., borborygmus, abdominal distention, and flatulence) and TPN support was therefore ceased.

**Table 1:** Nutrition support protocol for TPN and GIK group.

	POD 1	POD 2	POD 3	POD 4–7
TPN	TPN Fat: 110 g Carbohydrate: 200 g Amino acids: 41.25 g		TPN (at day) Fat: 110 g Carbohydrate: 200 g Amino acids: 41.25 g	EN Fat: 12.52 g Protein: 18.38 g Carbohydrate: 96.03 g Amino acids: 20 g I.V. Carbohydrate: 100 g
			EN (at night) Fat: 6.26 g Protein: 9.19 g Carbohydrate: 48 g	
	Calorie: 1951 kcal		Calorie: 2203 kcal	
GIK	I.V. Carbohydrate: 150 g		I.V. (at day) Carbohydrate: 150 g EN (at night) Fat: 6.26 g Protein: 9.19 g Carbohydrate: 48 g	
	Calorie: 600 kcal		Calorie: 851 kcal	Calorie: 1003 kcal

Notes: POD: postoperative days; TPN: Parenteral nutrition; EN: Enteral nutrition.

### Sample collection, processing and metabolome analysis

5 ml patients’ blood were collected and stored at −80 °C until analysis. Plasma cytokine measurement was conducted according to the manufacturer’s instructions by using the following analysis kit: Human IL-6 Quantikine ELISA Kit (R&D system, USA), Human IL-8 ELISA Kit, and Human Procalcitonin ELISA Kit (Abcam, USA). As for the metabolome analysis, the samples were thawed at room temperature, 100 μL of the plasma samples were then transferred into Centrifuge Tubes (1.5 mL) by pipette. All samples were extracted with 300 μL of methanol, and 10 μL of internal standard (3.0 mg/mL, DL-o-Chlorophenylalanine) was added. The samples were vortexed for the 30 s and centrifuged at 12000 rpm and 4 °C for 15 min. 200 μL of supernatant was transferred to the vial for LC-MS analysis. Samples were then added to Column (Hyper gold C18 (100 × 2.1 mm 1.9 μm)) and subjected to LC-MS (Thermo, Ultimate 3000LC, Q Exactive). The conditions were as follows: Chromatographic separation conditions: Column temperature: 40 °C; Flow rate: 0.35 mL/min; Mobile phase A: water + 5% acetonitrile + 0.1% formic acid; Mobile phase B: acetonitrile + 0.1% formic acid; Injection volume: 10 μL; Automatic injector temperature: 4 °C. Parameters for mass spectrum were as follows: ESI+: Heater Temp 300 °C; Sheath Gas Flow rate, 45arb; Aux Gas Flow Rate, 15 arb; Sweep Gas Flow Rate, 1arb; spray voltage, 3.0 KV; Capillary Temp, 350 °C; S-Lens RF Level, 30%. ESI-: Heater Temp 300 °C, Sheath Gas Flow rate, 45arb; Aux Gas Flow Rate, 15arb; Sweep Gas Flow Rate, 1arb; spray voltage, 3.2 KV; Capillary Temp,350 °C; S-Lens RF Level,60%.

### Statistical analysis and data presentation

The collected data of preoperative baseline and clinical outcomes were summarized and calculated for significance by *t*-test (two groups with homogeneity of variance), Mann-Whitney U test (two groups with the heterogeneity of variance), or Fisher’s exact test (contingency data) as appropriate. As for the metabolome, the data were first transformed to CDF files by Xcalibur (Version 2.2 SP1.63) and input into XCMS for peak picking, alignment, filtering, and filling. The data were normalized by peak-area normalization using Excel 2007, including Retention time (RT), MZ, Observations (samples), and peak intensity. 1735 features at (ESI+) ion mode and 1935 features at (ESI-) ion mode in this experiment, multivariate analysis (MVA) were performed on the data after normalization using SIMCA-P 13.0 software (Umetrics AB, Umea, Sweden). Principal component analysis (PCA) and orthogonal partial least squares discriminant analysis (OPLS-DA) were performed using SIMCA-P. The pathway analysis was conducted using MetaboAnalyst v.2.0 (www.metaboanalyst.ca). Additional manual update from the Kyoto Encyclopedia of Genes and Genomes (KEGG) database was conducted by two independent authors (Jiang Z. and Wen C.). Differential metabolites are defined by comparing mass-to-charge ratio or molecular mass in the Metlin metabolite database. The significance of each metabolites with difference was measured by Student *t* test. The resultant *P* values were corrected by Bonferroni correction and the *P* value of each metabolites during comparison were adjusted to decrease the risk of false-positive rate. The adjusted *P* value < 0.05 and variable importance in the projection (VIP) score >1 in the OPLS-DA model was selected as significantly differential molecular features as previously reported^19,20^.

## Results

### Clinical characteristics and outcomes of the study cohort

A total of 58 cases were enrolled in the trial, 6 cases dropped out due to the intraoperative change of surgery type and thus excluded from the following study. Patients were divided into GIK or TPN group based on their medical insurance type, and finally, 36 patients were allocated to the TPN group and 16 in the GIK group. Three blood samples for each patient were scheduled for collection, and 42 samples of POD 1, 40 samples from POD 4, and 36 samples from POD7 were finally collected. The preoperative clinical parameters, including nutrition status evaluated by Nutrition Risk Screening (NRS 2002), showed no differences between the two groups (Table 2).

**Table 2:** The baseline of 52 subjects included in the study.

	TPN (n = 32)	GIK (n = 16)	*P*
**Basic Characteristics**			
Age	62(54.5–66)	61(55–67)	0.52
Sex ratio (male/%)	15(46.8)	7(43.8)	1.0
BMI	22.8(20.3–25.9)	22(19.8–23.6)	0.68
**Comorbidities**			
CAD	4(12.5)	0	0.29
Hypertension	11(33.4)	6(37.5)	1.0
Diabetes mellitus	5(15.6)	6(37.5)	0.18
**Nutrition Status**			
NRS 2002	3.182(2–6)	3.211(2–5)	0.93
**Preoperative lab tests**			
Hemoglobin	110(95–129)	104(91–128)	0.92
White blood cell	8.66(5.35–11)	6.53(4.99–5.7)	0.52
Platelet	182(144–218)	188(135–251)	0.98
Total protein	59(54–70)	61(56–65)	0.72
Albumin	36(34–42)	37(36–40)	0.94
Total bilirubin	22.6(12.4–61.7)	16.9(12.4–64.9)	0.78
ALT	58(24–194)	46(24–62)	0.12
AST	53(21–101)	40(21–62)	0.24
Blood glucose	6.5(5.45–8.65)	6.4(5.5–8.8)	0.91
PCT	0.07(0.035–0.2)	0.048(0.02–0.25)	0.29
IL-6	3.04(2.42–4.70)	2.8(2–4.04)	0.11
IL-8	22.25(11.4–70.52)	18.2(10.6–43.95)	0.33

Notes: Data presented as number (percentage) or median (interquartile range). BMI: Body mass index; CAD: Coronary heart (artery) disease; ALT: Alanine aminotransferase; AST: Aspartate aminotransferase; PCT: Procalcitonin; *P*-value is calculated by t-test, Mann-Whitney U test or Fisher’s exact test as appropriate.

We summarized the clinical outcome of patients after the surgery, including intraoperative blood loss, transfusion, surgery time, gastrointestinal fistula, abdominal infection, abdominal hemorrhage, gastric emptying disorder, IL-6, IL-8, and PCT at each timepoint, postoperative hospital-stay, overall hospital-stay, percentage of perioperative weight loss and medical costs. We found TPN group showed decreased weight loss than GIK group while no other differences between the two groups were observed in these outcomes (Table 3), indicating the TPN support, compared with the GIK group, could reduce the body consumption but did not increases postoperative complications.

**Table 3:** Clinical outcomes of 52 subjects included in the study.

	TPN (n = 32)	GIK (n = 16)	*P*
Intraoperative Blood Loss (ml)	300 (200–400)	200 (200–400)	0.9727
Transfusion (Event)	3.03% (1/32)	0 (0/16)	0.2453
Surgery Time (min)	180 (150.5–200)	180.8 (120–221)	0.8665
Enteral Nutrition Initiation (day)	3 (2.5–5)	3 (3–4)	0.7358
Gastrointestinal Fistula (n)	6.25% (2/32)	0 (0/16)	0.5461
Abdominal Hemorrhage (n)	3.13% (1/32)	6.25% (1/16)	>0.9999
Abdominal Infection (n)	18.75% (6/32)	6.25% (1/16)	0.3983
Blood Stream Infection (n)	9.38% (3/32)	0% (0/16)	0.5412
Gastric Emptying Dysfunction (n)	4.17% (2/32)	0 (0/16)	0.5461
Postoperative Hospital Stay (day)	9 (6.5–17)	7 (6–12)	0.0818
Overall Hospital Stay (day)	13 (10.5–20)	12 (9–15)	0.1154
Perioperative Weight Loss (%)	11.6 (10–14)	9.28 (7–11)	0.0326
Medical Costs (RMB: yuan)	66640 (60970–80870)	59230 (47510–73060)	0.1137
**PCT (pg/ml)**			
(POD 4)	57.23(45.93–120.2)	113.1(45.01–197)	0.1379
(POD 7)	39.74(28.64–69.87)	70.73(51.1–79.22)	0.2863
**IL-6 (pg/ml)**			
(POD 4)	42.65 (13.74–51.65)	33.52 (11.65–24.97)	0.8685
(POD 7)	20.93 (8.88–21.98)	26.05 (8.56–36.17)	0.9289
**IL-8 (pg/ml)**			
(POD 4)	7.95(4.49–14.12)	4.21(1.59–9.45)	0.1996
(POD 7)	5.71(2.72–9.07)	2.72(1.22–13.93)	0.8896

Notes: Data presented as number (percentage) or median (interquartile range). PCT: Procalcitonin; *P*-value is calculated by t-test, Mann-Whitney U test, or Fisher’s exact test as appropriate.

### Metabolome characterisation

**Figure 2:**
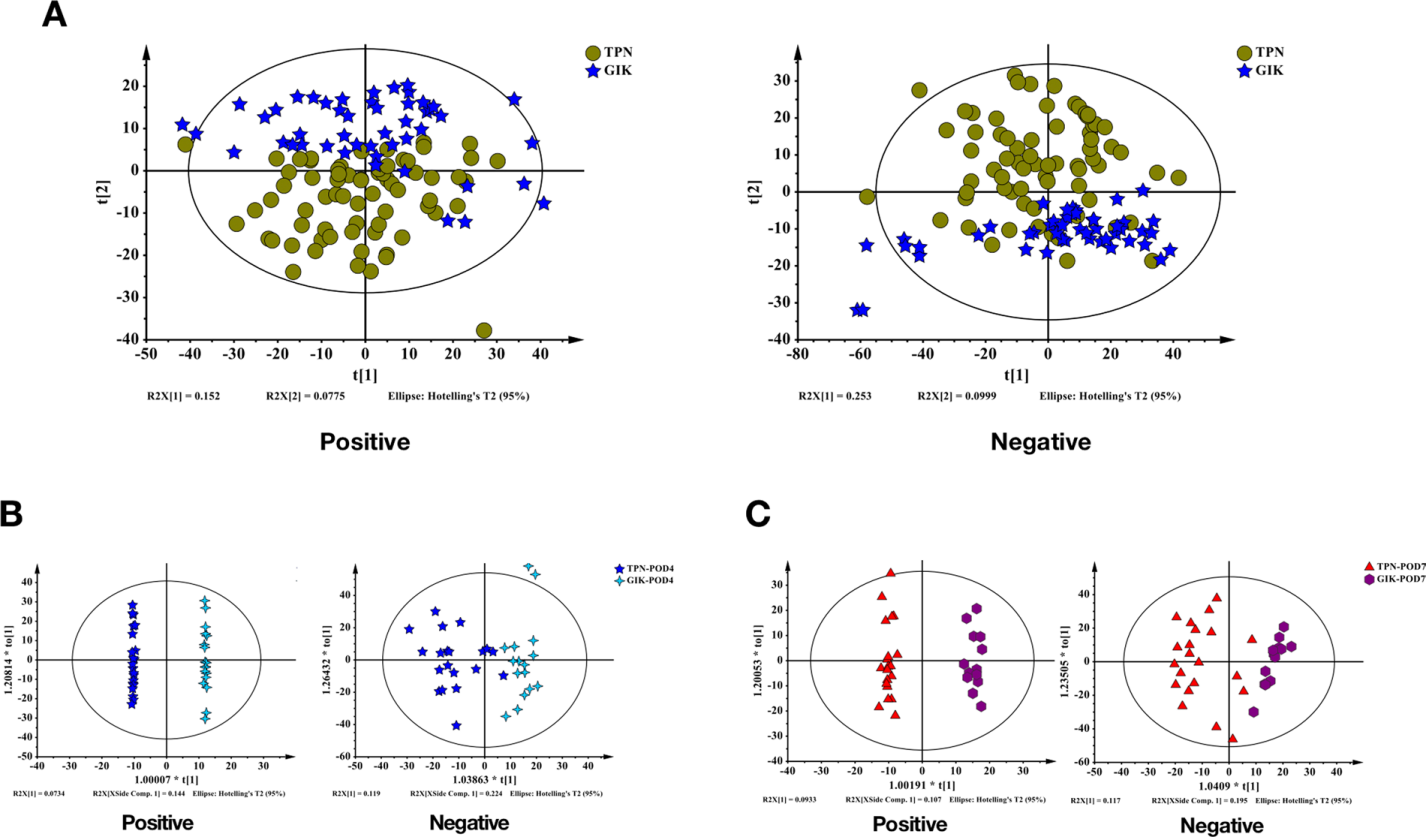
Multivariate statistical analysis of plasma metabolome between TPN and GIK group. (**A,B**) Principle component analysis (PCA) generated from positive and negative modes of total samples in TPN and GIK group (n = 118). (**B,C**) Orthogonal partial least squares discriminant analysis (OPLS-DA) of TPN and GIK groups on POD 4 (TPN: n = 22; GIK: n = 18) and 7 (TPN: n = 21; GIK: n = 15) compared to POD 1 (TPN: n = 27; GIK: n = 15).

To distinguish the differences of metabolism between GIK and TPN, we conducted metabolome analysis on all the samples. Principal component analysis (PCA) was used for the analysis of TPN and GIK groups, a total of 19 principal components were obtained in the positive mode (one sample excluded due to abnormal data) and 15 in negative mode (nine sample excluded due to abnormal data) (R^2^X = 0.684, Q^2^ = 0.467) (Fig. 2A). The results showed that significant differences existed between the two groups in metabolic profiling.

**Figure 3:**
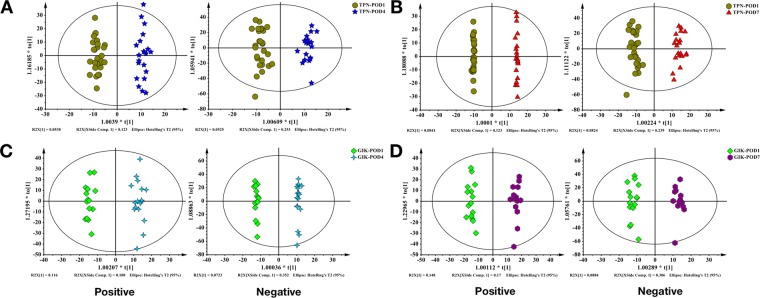
Multivariate statistical analysis of plasma metabolome between POD 4 and POD 7 in TPN and GIK groups. (**A,B**) OPLS-DA of TPN group in POD 4 (POD1: n = 27; POD4: n = 22) and POD 7 (POD7: n = 21); (**C,D**) OPLS-DA of GIK group in POD 4 (POD1: n = 15; POD4: n = 18) and POD 7 (POD7: n = 15).

We also analyzed the differences between two groups at POD 4 (Fig. 2B) and POD 7 (Fig. 2C), or difference between time points (POD 4 to POD 1 and POD 7 to POD 1) (Fig. 3A–D) in each group by orthogonal partial least squares discriminant analysis (OPLS-DA). The results also show the difference between comparisons. Besides, no over-fitting was observed in all comparisons according to the results of permutation tests (Supplemental Fig. 1).

**Figure 4:**
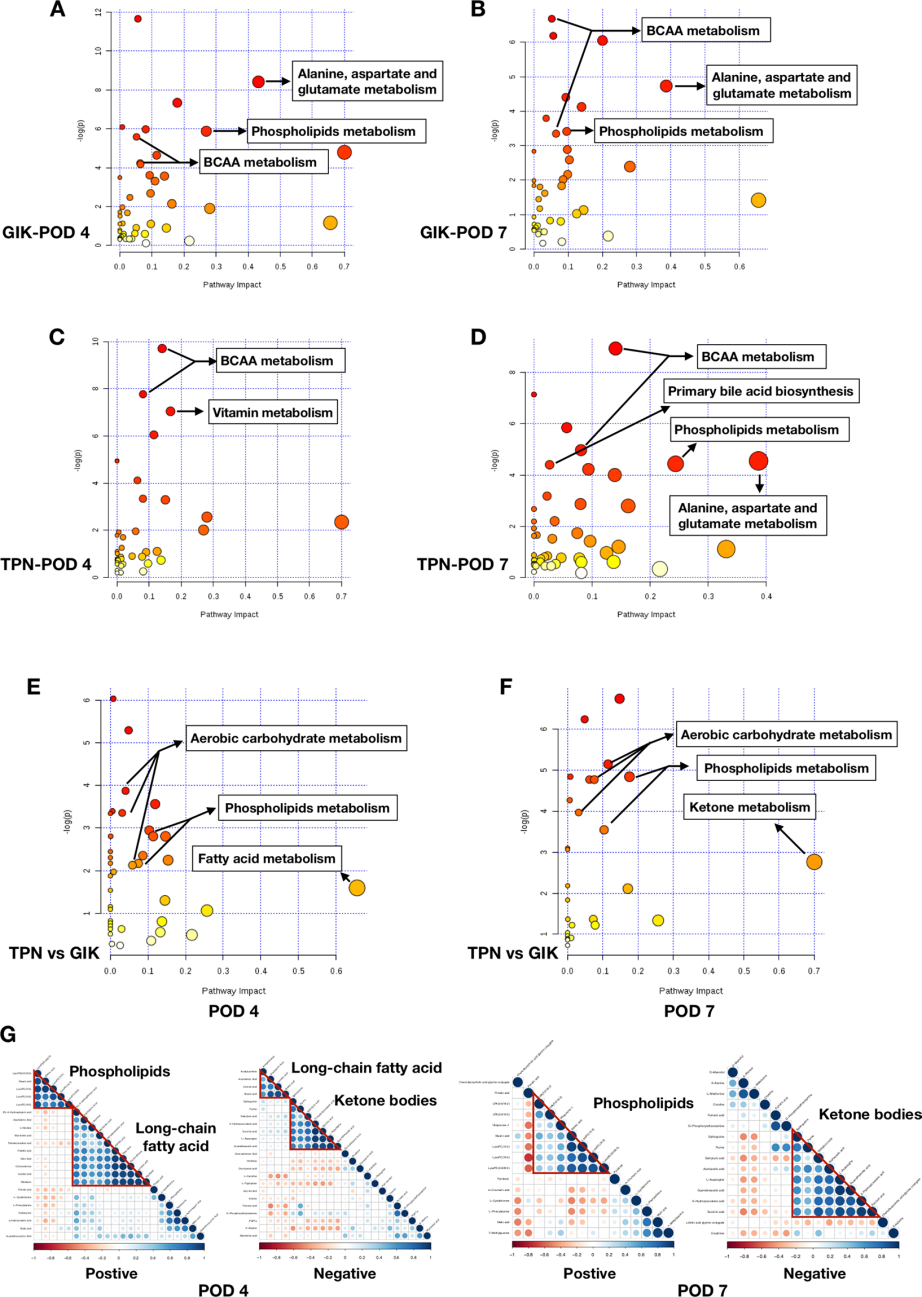
Metabolic pathway analysis of identified differential metabolites in TPN and GIK groups. (**A,B**) Metabolic pathway analysis of POD 4 compared to POD 1 (**A**) and POD 7 compared to POD 1 (**B**) in the GIK group. (**C,D**) Metabolic pathway analysis of POD 4 compared to POD 1 (**A**) and POD 7 compared to POD 1 (**B**) in the TPN group. (**E**,**F**) Metabolic pathway analysis of TPN compared to GIK group on POD 4 (**E**) and POD 7 (**F**). Node color based on p-value and node radius determined based on pathway impact values. G: Differential metabolites correlation in Positive and negative modes. Node color based on increased or decreased alteration and node color depth and size based on metabolites correlation.

We then focused on the differentially represented metabolites. After identifying the differential metabolites by featured criteria (Method; Supplemental Table 1, Supplemental Dataset), we analyzed the metabolic pathways related to the identified metabolites using MetaboAnalyst v.2.0 software and allocated metabolism according to KEGG database. Firstly, in order to clarify the metabolic transition in TPN and GIK group, we analyzed the alteration in POD 4 (Fig. 4A,C) and POD 7 (Fig. 4B,D) compared with POD 1 in each group respectively. In POD 4, a significant elevation of branched-chain amino acids (BCAA) metabolism was observed in the TPN group combined with vitamin metabolism (Fig. 4C). However, in the GIK group, BCAA metabolism remained less significant, whereas alanine, aspartate, and glutamate metabolism was more impactive and enhanced (Fig. 4C). In POD 7, metabolism in the GIK group remained similar to POD 4, except for the elevation of BCAA metabolism (Fig. 4B), whereas, in the TPN group, phospholipid and BCAA metabolism became more significant and impactive, and primary bile acid biosynthesis was elevated (Fig. 4D). Taken together, these results suggested that the TPN group showed more active anabolic protein metabolism (e.g., muscle protein synthesis based on BCAA metabolism) and a more significant recovery of lipid metabolism (Fig. 4).

We then investigated the differences between TPN and the GIK group on POD 4 and 7. Firstly, in POD 4, more significant aerobic carbohydrate metabolism of tricarboxylic acid (TCA) cycle and related pathways (butanoate and propanoate metabolism) was observed in the TPN group (Fig. 4E). Moreover, phospholipids and fatty acid metabolism are also impactive in TPN (Fig. 4E). These results were further enhanced to be more impactive and elevated in POD 7, and ketone metabolism was also enhanced (Fig. 4F). We then analyzed the correlation of metabolites to evaluate the differential metabolites in related metabolic pathways further. The results were consistent with the pathway enrichment analysis that phospholipid, ketone bodies, and fatty acid metabolism were significantly elevated in the TPN group (Fig. 4G). These results indicated that the TPN group helped the recovery of lipid metabolism and suggested that anabolic metabolism and tissue repair may be more active due to the increased phospholipids metabolism.

### Metabolite ingenuity pathway analysis (ipa) and network construction

**Figure 5:**
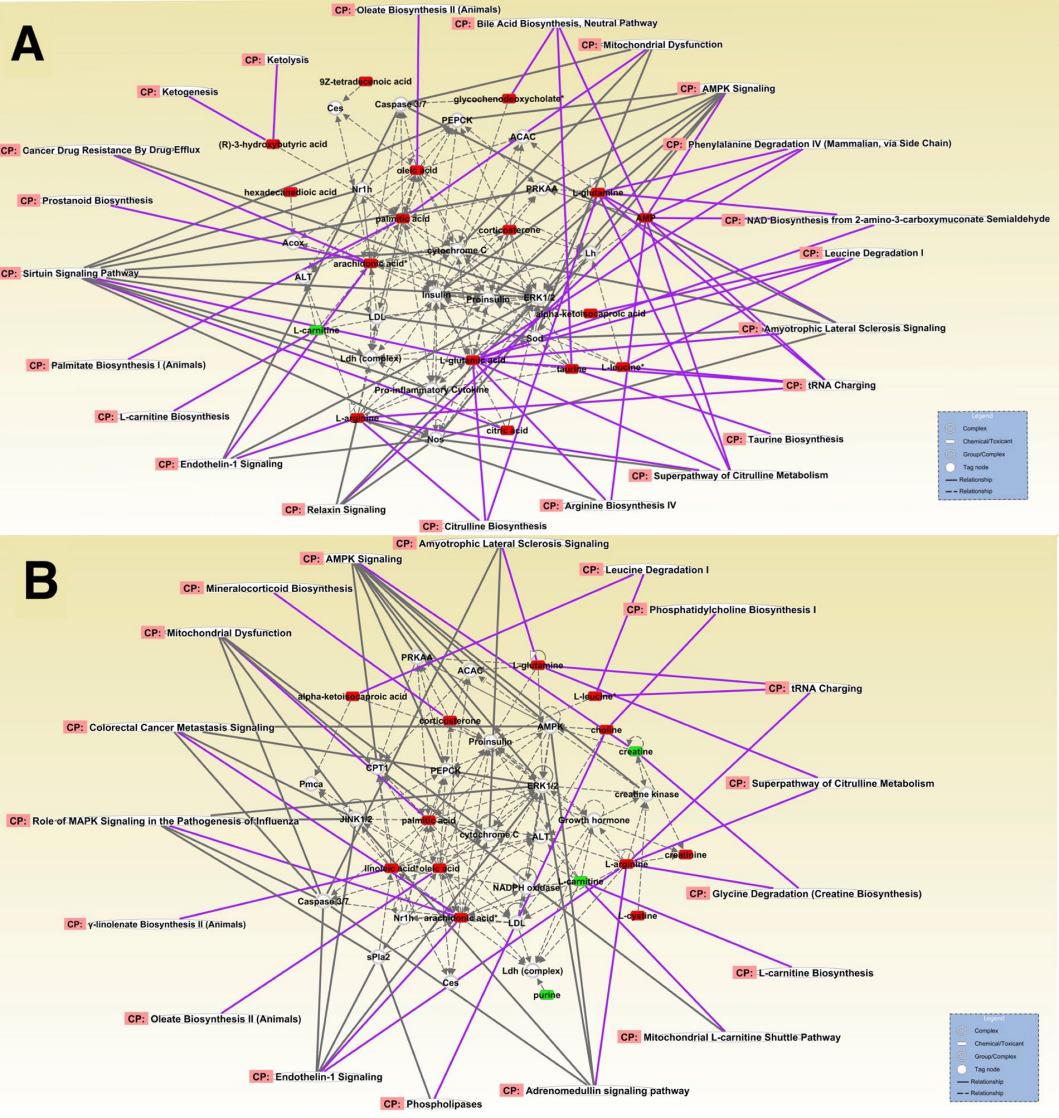
Ingenuity Pathway Analysis (IPA) of differential metabolites of POD7 in GIK and TPN group, respectively. (**A,B**) Analysis based on the comparison in POD 7 compared to POD 1 of TPN (**A**) and GIK (**B**) group, respectively. Rectangle represented elevated (red) or decreased (green) metabolites. The peripheral represented metabolic pathways. The central circle represented signaling complex or group. The full and dotted line represented direct (full) and indirect (dotted) correlation to signaling. The full line in purple represented the direct correlation between metabolic pathways and metabolites.

To further overview how differential metabolites interact with metabolic pathway and signaling, we conducted the integrated IPA to mine the metabolome data further. We first analyzed the network of POD 7 compared with POD 1 in the TPN group (Fig. 5A) and GIK group (Fig. 5B), respectively. The result showed consistency with the previous pathway enrichment that amino acids and lipid metabolism were elevated in both two groups, whereas more diverse metabolism type and metabolites were observed in TPN group, such as more diverse long-chain fatty acid (hexadecanoic and tetradecenoic acid), more active bile acid and ketone body metabolism, more various type of amino acids metabolism centered by glutamate or glutamine, and increased citric acid were observed in TPN group (Fig. 5A). Interestingly, we also notice that insulin signaling was elevated as a central signal regulator in the TPN group (Fig. 5A), which further indicates the anabolic metabolism in lipid and amino acids. While, on the contrary, decreased creatine and elevated creatinine observed in the GIK group (Fig. 5B) suggested the increased deamination of amino acid and possible energy consumption generated from amino acids.

**Figure 6:**
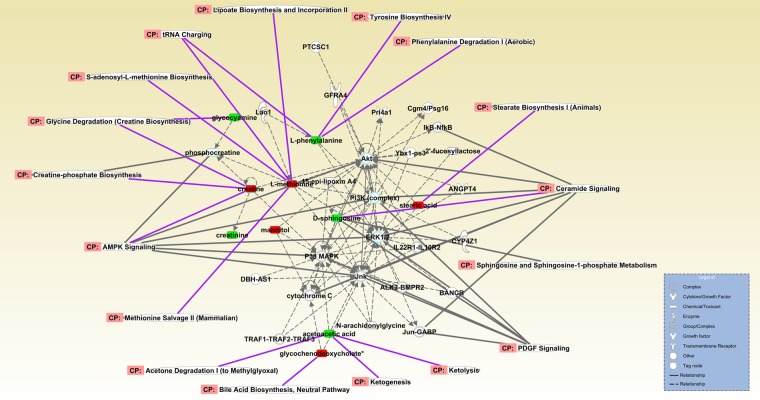
Ingenuity Pathway Analysis (IPA) of differential metabolites in POD 7 between GIK and TPN. Analysis based on comparison of TPN versus GIK in POD 7. Merged network combining primary signaling related to the differential metabolites expressed in the ingenuity pathway analysis. Rectangle represented elevated (red) or decreased (green) metabolites. The peripheral represented metabolic pathways. The central circle represented signaling complex or group. The full and dotted line represented direct (full) and indirect (dotted) correlation to signaling. The full line in purple represented the direct correlation between metabolic pathways and metabolites.

As for the differences comparing two groups in POD 7, in accordance with the pathway enrichment (Fig. 4), the integrated network indicated the elevation in ketone body, fatty acid and amino acid metabolism (Fig. 6), for example, ketone body mediated by acetoacetic acid, fatty acid-mediated by stearic acid and amino acids mediated by phenylalanine and creatine.

Metabolites interact with intracellular or transcellular signaling and molecules^21–23^. Through the network of differential metabolites, we found AMPK signaling acted as the central role in regulating most of the differential metabolites (Figs. 5, 6), suggesting the postoperative metabolic transition mainly associated with AMPK and related signaling.

## Discussion

Guidelines recommended the application of enteral nutrition within 48 hours postoperative for critical care patients^8,9^. However, for patients undergoing major abdominal surgery like pancreaticoduodenectomy, EN within 24 to 48 hours may not be applied with enough nutrients due to postoperative gastrointestinal dysfunction or unstable clinical status^10,11,24^. Therefore, clinical situation, like enrolled patients in GIK group in the present study, is usually mixed that some patients can be applied with EN within 48 hours while some may not be able to tolerant due to gastrointestinal risks. On the other hand, catabolism activated by surgical trauma, inflammation, or oxidative stress at the early postoperative period deteriorate metabolic homeostasis and, therefore, require additional nutrients and calories^25–27^. In extreme cases like sepsis, blockage of lipid oxidation and catabolism of amino acid deteriorate the homeostasis and eventually lead to poor prognosis or death^15,26^. Therefore, decreased gluconeogenesis from amino acid and utilization of lipid in the present study suggested the TPN support may help the metabolic transition to homeostasis and thus enhance the patients’ recovery^25,28^.

Total parenteral nutrition (TPN) provides nutrients of amino acids, lipids, and vitamins, which may provide additional benefits for the transition of metabolism and help the body be tolerant of following EN^5,7^, while the risk of blood glucose disorder or infection are significant concerns for TPN application^7,28^. In the present study, though the sample size is relatively small, clinical outcomes between the two groups indicated no increased infection or other complications. This may be due to the application of alanyl-glutamine added in the TPN group that was reported to decrease nutrition-related infection^7^. Another study reported more convincing results of similar early TPN support that enhanced the recovery of pancreatic surgical patients in plenty of clinical indexes^13^, which is also echoed by the results of the reduction of perioperative weight loss in TPN group that indicated the benefits of approaches of TPN + EN. However, those studies provided no information on how metabolism changes or in what mechanism that helped the recovery of patients. Therefore, in comparison, we monitored the metabolic transition and evaluated their possible metabolic benefits.

The postoperative metabolic transition has been proved to regulate immunity, anti-oxidant activity, and efficient energy metabolism^11,12,15,25^. In analyzing the metabolic transition, we noticed that the elevation of phospholipid metabolism occurred in both two groups, whereas TPN was more significant and impactive (Fig. 4). This was further proved by the comparison between TPN versus GIK in POD 7 where differences in lipid metabolism were further evident and is in accordance with the results of a previous research that the re-establishment of phospholipids and fatty acid metabolism was related to better outcome and decreased risks of mortality, whereas breakdown of lipid metabolism, especially the elevated carnitine esters were related to inevitable death^15^. Taken together, our results suggested that early TPN support may lead to enhanced generation of ketone body, fatty acid utilization, and aerobic carbohydrate metabolism re-establishment and help transfer to the “high-efficiency” or more beneficial metabolism for postoperative recovery^11,26^.

Another notice lies in the metabolism of amino acids. We observed that alanine, aspartate, and glutamate metabolism was primary metabolism of amino acids, and BCAA remains a low level in GIK at POD 4, while in the TPN group, BCAA is the most significant metabolism together with vitamin. Indeed, surgical trauma may cause a shift of metabolism that uses glycolysis as a significant energy source and quickly exhausts glucose storage^4,29^. Moreover, at this time, lipid oxidation is usually blocked^4,29^. Therefore, gluconeogenesis from amino acids becomes a significant source of energy supply, and muscular tissue is firstly mobilized^29–31^. In this case, BCAAs in muscles act as a source of nitrogen for the synthesis of alanine and glutamine, which was then released to blood as gluconeogenic substrates^29^. The difference between GIK and TPN group reflected that the sufficient calorie supply from the TPN at early postoperative periods is helpful to the transition from glycolysis and gluconeogenesis to anabolic metabolism, which is crucial for preserving muscular homeostasis and other organ functions. Thus, these results indicated that TPN support with sufficient nutrients and calories is beneficial for pancreatic patients in restoring energy supply and preserve protein storage.

Nutrient metabolism linked to various cell signaling and pathway regulations^21–23^. Identifying crucial regulators or signals of metabolic transition is essential to understand the critical components of nutrition support. Companied with enhanced lipid metabolism, AMPK signal, together with sirtuin signaling, is enriched in the TPN group (Figs. 6, 5). As a critical regulator for lipid-glucose metabolism^32–34^, AMPK signal has been proved related to metabolic diseases like obesity, diabetes, and cardiovascular diseases^23,32^. The enrichment of AMPK signaling in our study partly explained why lipid metabolism was active, and homeostasis, including ketone body utilization, fatty acid oxidation, and phospholipid synthesis, were attained^23,32,35^. AMPK and sirtuin signaling are reciprocal interacted^23,32^ in lipid regulation, and more diverse long-chain fatty acid was observed mainly in metabolic transition in the TPN group (Fig. 5A). Also, the result implicated that metabolic transition may be initiated from AMPK signaling and thus suggested possible targets for a metabolic breakdown of lipid in critical patients. Another notice lies in ceramide signaling that enriched in the network (Fig. 6). Ceramide signaling mainly participates in inflammatory responses and many other cellular functions^21,36^. Especially sphingosine, a metabolite decreased after TPN support, had been proved relating to pulmonary inflammation, which interacted with NF-kB to initiate pro-inflammatory responses^36^. A decreased level of sphingosine indicated the resolving of postoperative inflammation and oxidative stress, and enhanced tissue repair and wound healing^21,36^.

We also constructed a possible connected metabolic pathway that TPN support altered most according to the KEGG database (Supplemental Fig. 2). We believe a possible theory might be that the early TPN support provided sufficient nutrition and enhanced the synthetic amino acids metabolism, which enhanced the biosynthesis of creatine and phosphocreatine that further activates AMPK and lipid metabolism^29,37^. As a result, the recovered positive energy balance promoted anabolic and aerobic metabolism^15,22^, which helped the patients’ postoperative adaptation and recovery.

Finally, there are also some limits to the present study. As this is a single-center, a small sample-sized study that only investigated nutrition support of TPN + EN, we do not know whether the conclusion could be extended to other general surgical patients or functions with similar benefits. Also, the allocation according to patients’ insurance type contains potential heterogeneity of the sample. Future researches in other nutrition support performed in more various surgery types with multi-centered evaluation should be carried out to confirm the best option or most important component of nutrition support. Moreover, the plasma metabolism profile reflects the general metabolism conditions, and it is hard to identify metabolic changes in specific organs (such as liver or kidney) or metabolism in specific cells (such as neutrophils or monocytes/macrophages). More detailed information about how nutrition support influences the metabolism, and functions in specific organs or tissues, are still needed to confirm a more direct and applicable conclusion. Last but not least, recent insights focusing on the effects of nutrition support on immunology and inflammation have attained promising results^7,25^. Though the inflammatory cytokines showed no differences, more investigations on the nutritional therapy on tumor immunology and systematic inflammation might be an intriguing direction for critical patients.

## Conclusion

The present study found that the early postoperative TPN support could reduce the weight loss and promote the recovery of the metabolism for patients undergoing pancreaticoduodenectomy, especially in promoting positive nitrogen balance and lipid metabolism, indicating the necessity and benefits of parenteral nutrition in the early stage of postoperative periods in pancreatic surgical patients.

## Supplementary information

Dataset 1-6

Supplemental Figures

Supplemental table 1

## Data Availability

The datasets generated during and/or analysed during the current study are available in the zenodo repository, [https://zenodo.org/record/3550291#.XddGl5MzaX1].
