# Supplementary figures and images for: Plasma metabolomics of early parenteral nutrition followed with enteral nutrition in pancreatic surgery patients
Source: Sci Rep. 2019 Dec 11;9:18846. doi: 10.1038/s41598-019-55440-z (PMC6906312; doi:10.1038/s41598-019-55440-z)

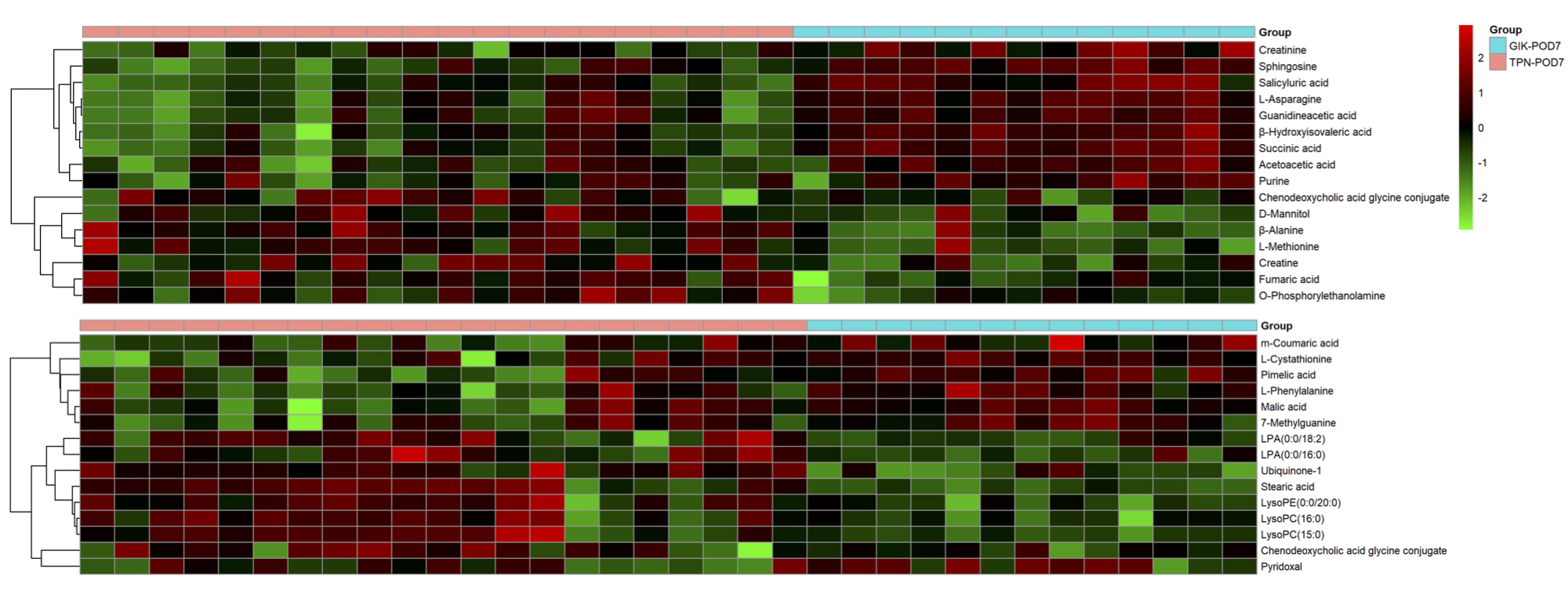

Supplement: Supplementary file 1 — Dataset 1-6 [file 41598_2019_55440_MOESM1_ESM.zip › Supplemental dataset/Supplemental dataset 6.jpg]

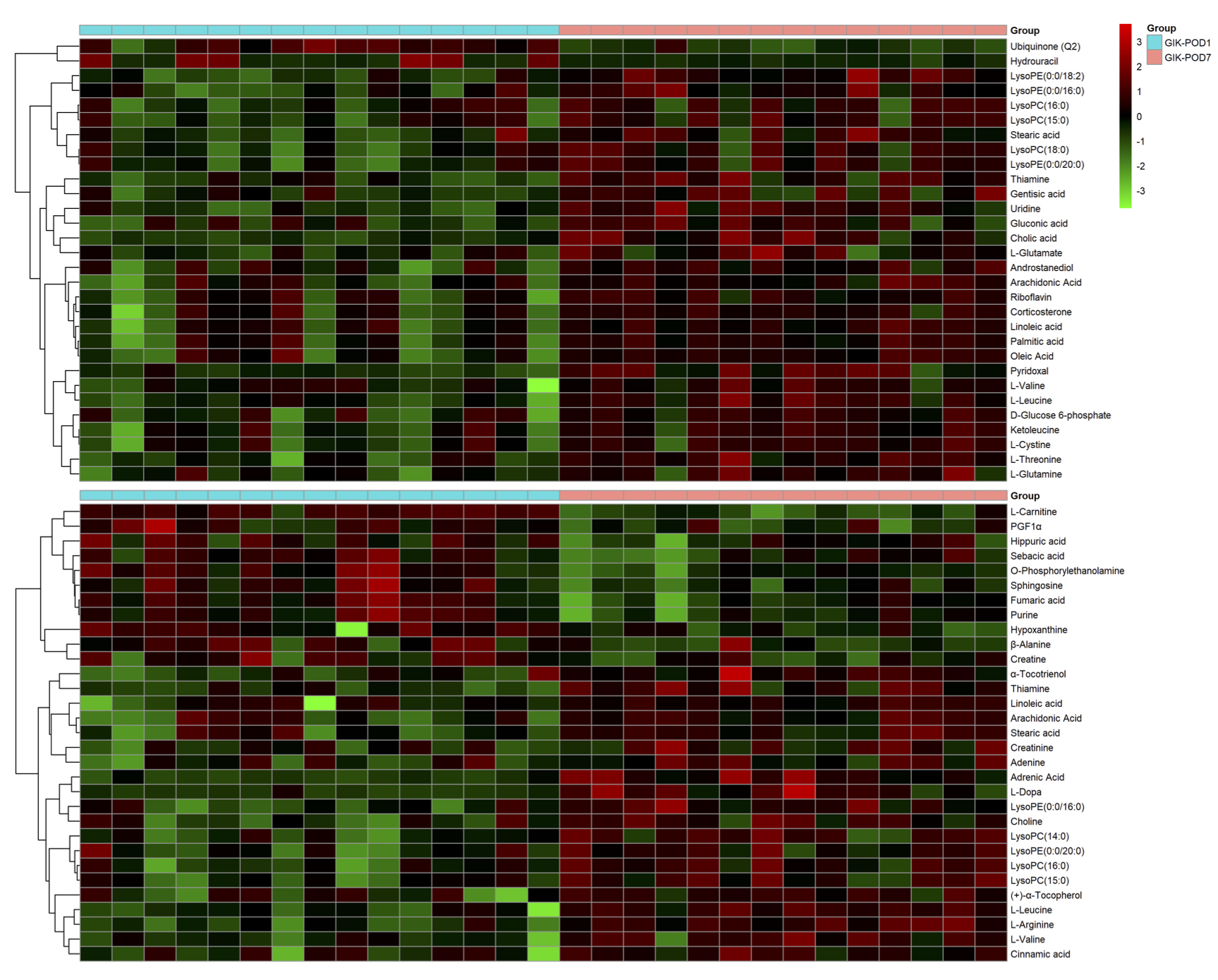

Supplement: Supplementary file 1 — Dataset 1-6 [file 41598_2019_55440_MOESM1_ESM.zip › Supplemental dataset/Supplemental dataset 4.jpg]

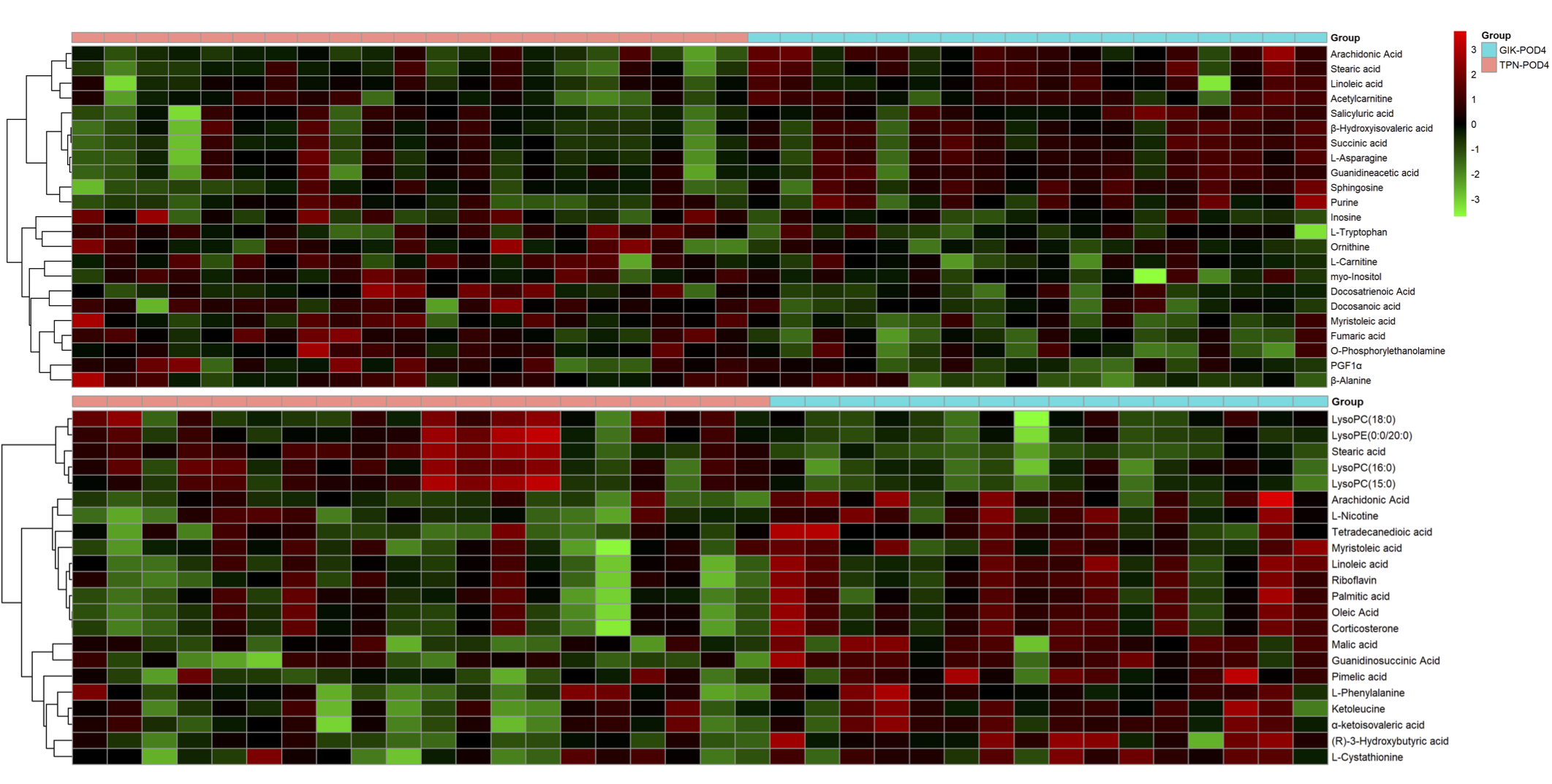

Supplement: Supplementary file 1 — Dataset 1-6 [file 41598_2019_55440_MOESM1_ESM.zip › Supplemental dataset/Supplemental dataset 5.jpg]

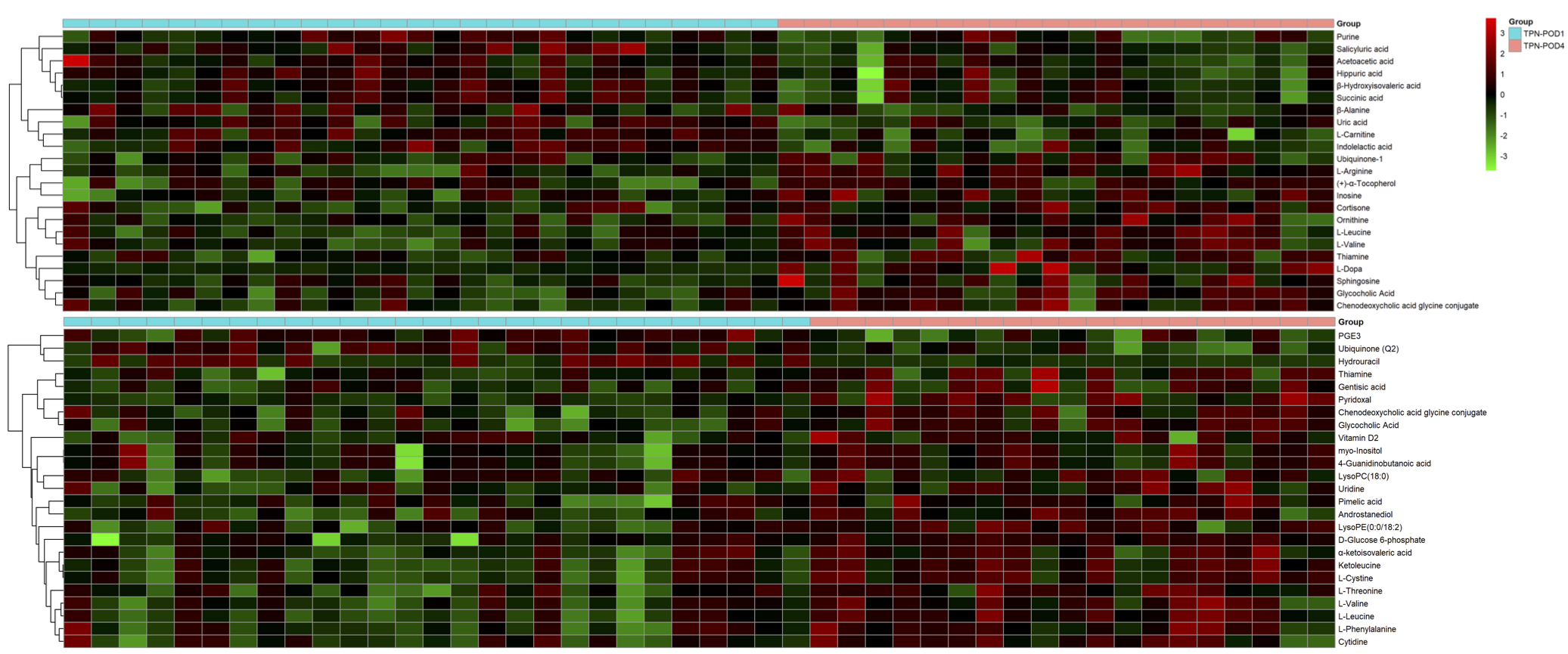

Supplement: Supplementary file 1 — Dataset 1-6 [file 41598_2019_55440_MOESM1_ESM.zip › Supplemental dataset/Supplemental dataset 1.jpg]

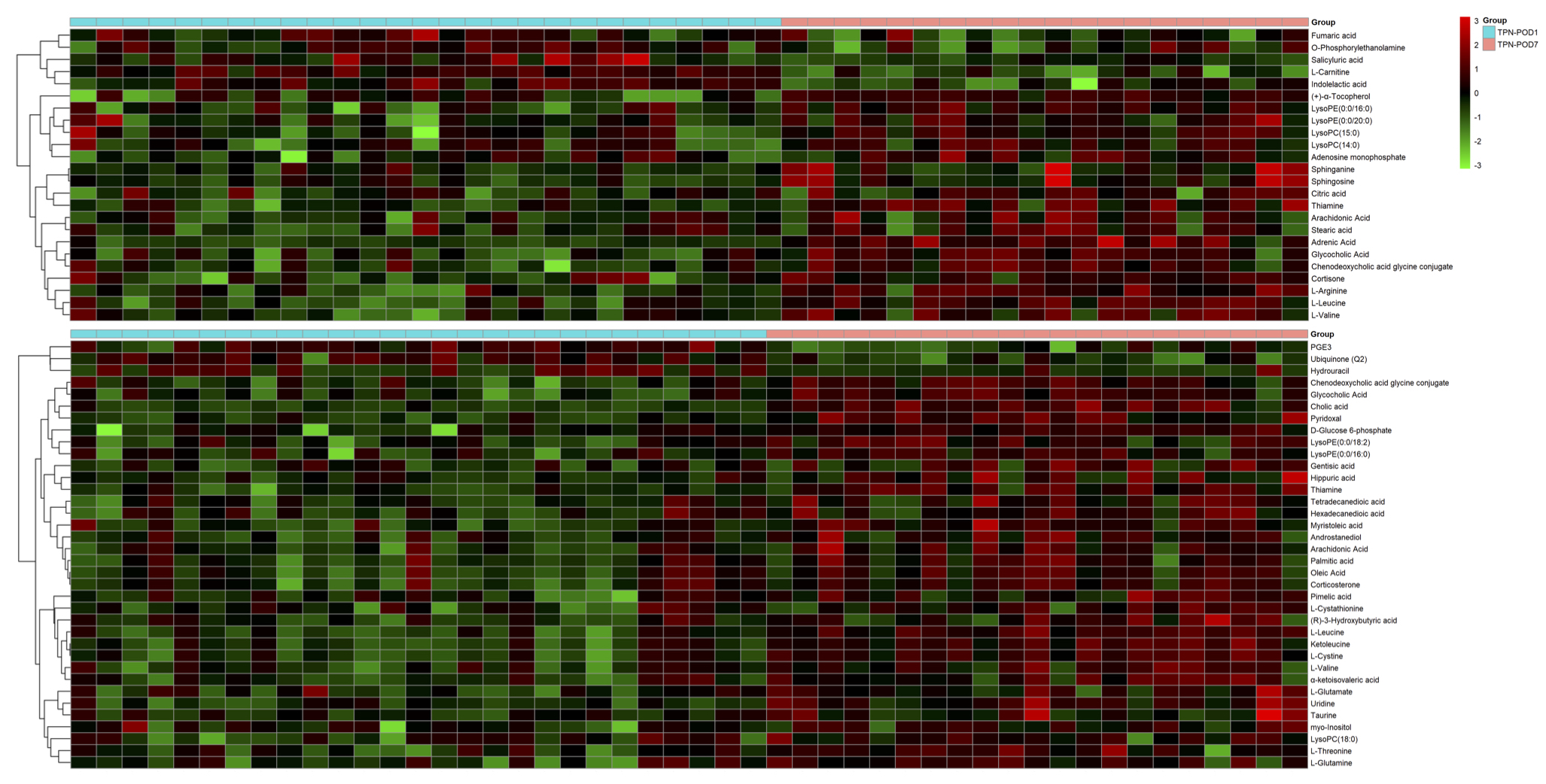

Supplement: Supplementary file 1 — Dataset 1-6 [file 41598_2019_55440_MOESM1_ESM.zip › Supplemental dataset/Supplemental dataset 2.jpg]

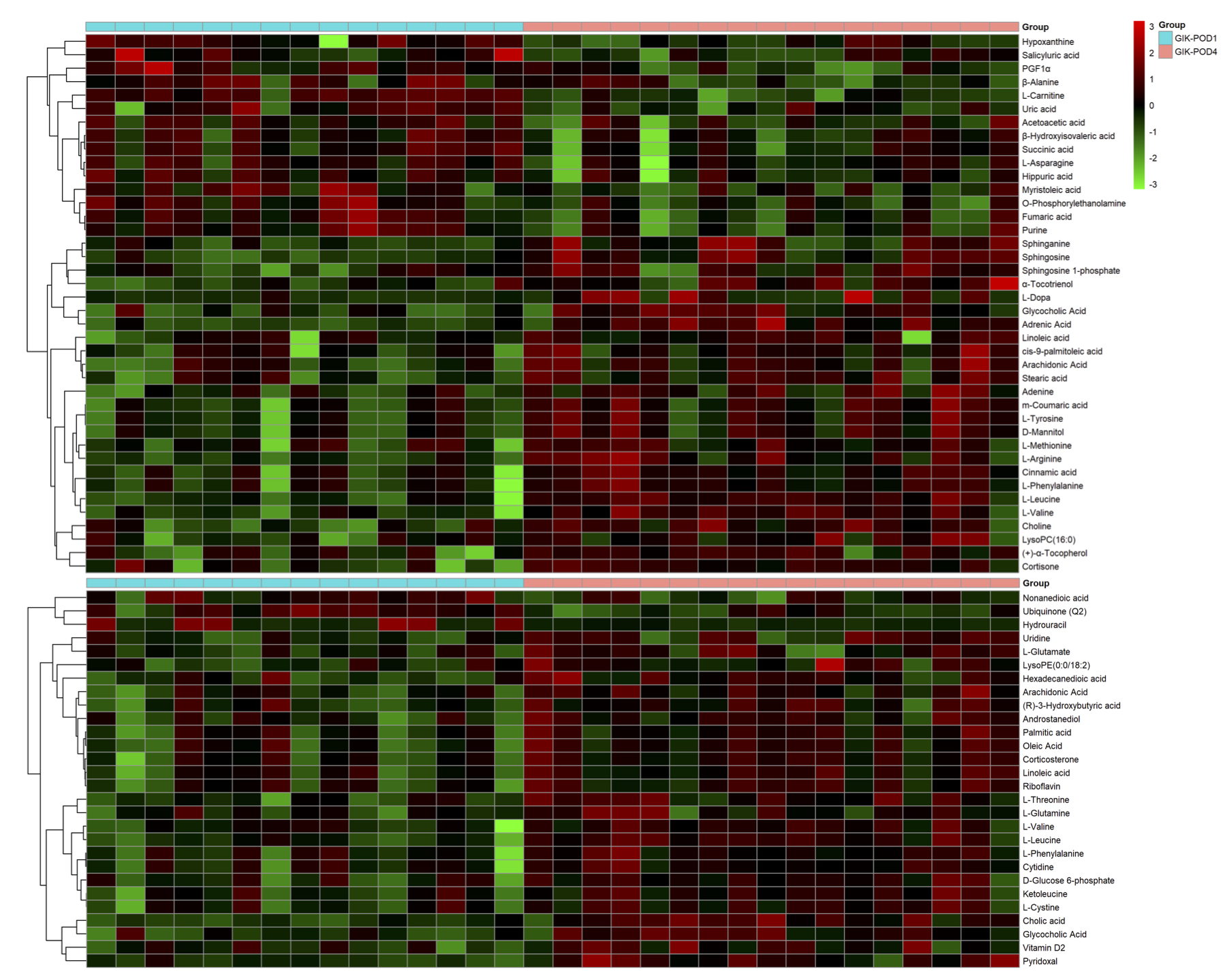

Supplement: Supplementary file 1 — Dataset 1-6 [file 41598_2019_55440_MOESM1_ESM.zip › Supplemental dataset/Supplemental dataset 3.jpg]
