# Supplementary material for: Plasma metabolomics of early parenteral nutrition followed with enteral nutrition in pancreatic surgery patients
Source: Sci Rep. 2019 Dec 11;9:18846. doi: 10.1038/s41598-019-55440-z (PMC6906312; doi:10.1038/s41598-019-55440-z)
Supplement: Supplementary file 2 — Supplemental Figures [file 41598_2019_55440_MOESM2_ESM.pdf]

Plasma metabolomics of early parenteral nutrition followed with enteral nutrition in pancreatic surgery patients

Zhengyu Jiang[1, §], M.D., Cen Wen[1, §], M.D., Changli Wang[1, §], M.D., Zhenzhen Zhao[1], M.D., Lulong Bo[1], M.D., Xiaojian Wan[1, ¶], M.D., Xiaoming Deng[1, ¶], M.D., Ph.D.

[1] Faculty of Anesthesiology, Changhai Hospital, Second Military Medical University, 200433, Shanghai, China

[§] Equal contributions

[¶] Corresponding authors

Supplemental Figure 1. Permutation tests in positive or negative mode of comparisons in the study.

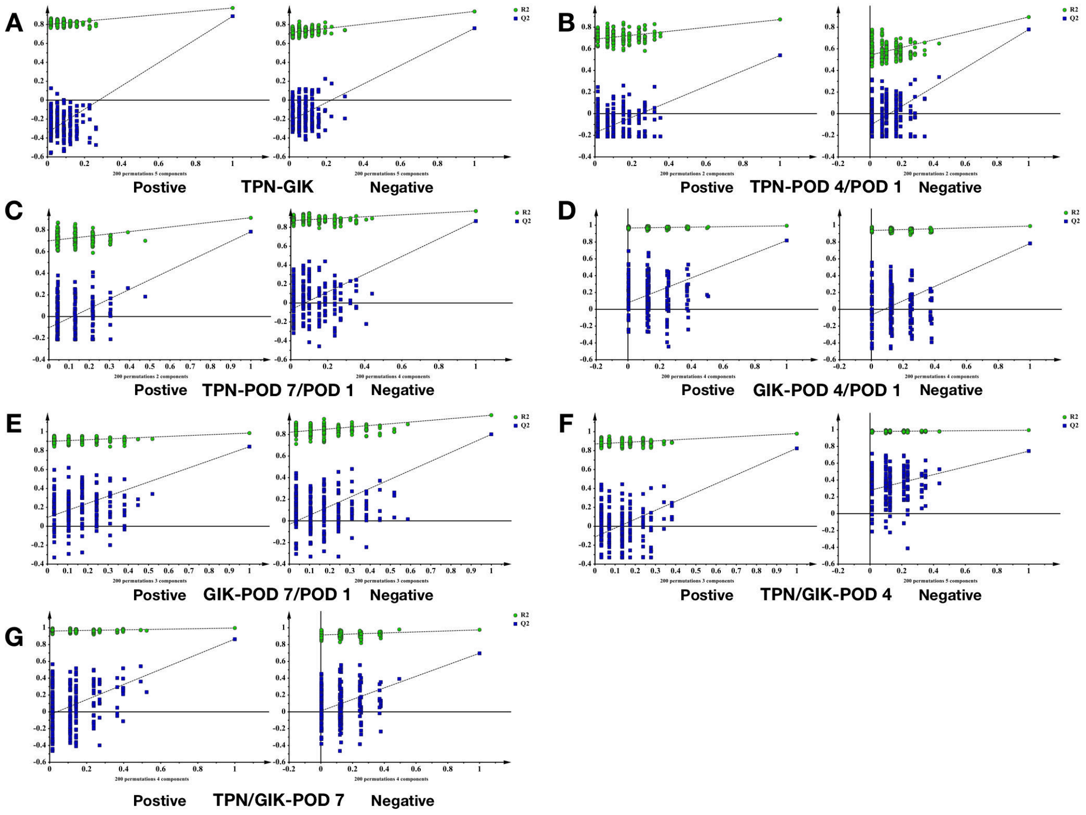

A: Permutation tests of all samples of TPN and GIK group. B-E: Permutation tests of TPN group (B,C) in POD 4 (B) or POD 7 (C) compared to POD 1, or GIK group (D,E) in POD 4 (D) or POD 7 (E) compared to POD 1. F,G: Permutation tests of TPN compared to GIK group in POD 4 (F) and 7 (G).

Supplemental Figure 2. Major altered pathway after early PN support followed with EN in patients underwent PD.

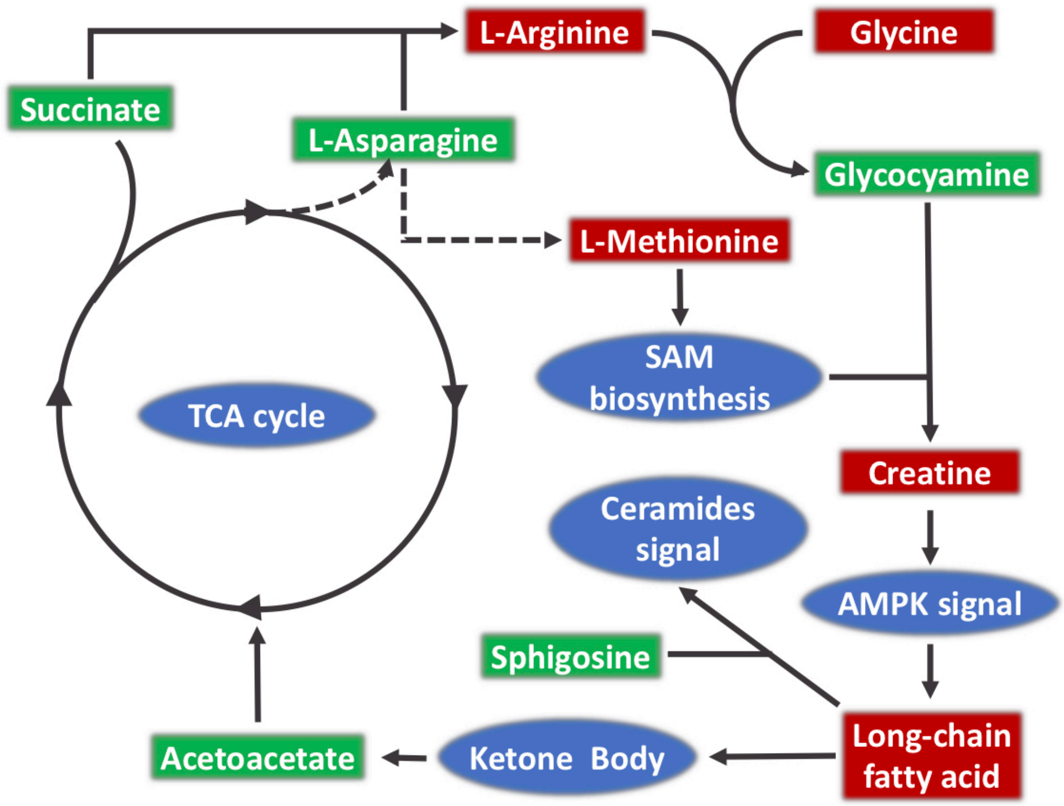

Early PN support provided sufficient nutrition and enhanced the synthetic amino acids metabolism, which enhanced biosynthesis of creatine and phosphocreatine that further activates AMPK and lipid metabolism. As a result, the recovered positive energy balance promoted anabolic and aerobic metabolism, which helped the patients' postoperative adaptation and recovery. Elevated (red) and decreased (green) metabolites of TPN compared to GIK in POD 7 according to IPA analysis. Oval in blue represented major signal or metabolic pathways that influenced by TPN support. Full and dotted line represented direct (full) and indirect (dotted) correlation to metabolites or metabolic pathways.

Supplemental Dataset. Heatmap of differential metabolites of comparison in the study. The differential metabolites with fold changes (red: elevation; green: decreasing) are presented by heatmap in Supplemental Dataset 1 (POD 4/POD1 in TPN), Dataset 2 (POD7/POD1 in TPN), Dataset 3 (POD4/POD1 in GIK), Dataset 4 (POD7/POD1 in GIK), Dataset 5 (TPN versus GIK in POD4) and Dataset 6 (TPN versus GIK in POD7).
