# Supplementary material for: Plasma metabolomics of early parenteral nutrition followed with enteral nutrition in pancreatic surgery patients
Source: Sci Rep. 2019 Dec 11;9:18846. doi: 10.1038/s41598-019-55440-z (PMC6906312; doi:10.1038/s41598-019-55440-z)
Supplement: Supplementary file 3 — Supplemental table 1 [file 41598_2019_55440_MOESM3_ESM.docx]

**Plasma metabolomics of early parenteral nutrition followed with enteral nutrition in pancreatic surgery patients**

Zhengyu Jiang^1, §^, M.D., Cen Wen^1, §^, M.D., Changli Wang^1, §^, M.D., Zhenzhen Zhao^1^, M.D., Lulong Bo^1^, M.D., Xiaojian Wan^1, ¶^, M.D., Xiaoming Deng^1, ¶^, M.D., Ph.D.

^1^ Faculty of Anesthesiology, Changhai Hospital, Second Military Medical University, 200433, Shanghai, China

^§^ Equal contributions

^¶^ Corresponding authors

**Supporting Table 1.** Differential metabolites list of comparisons in the study.

**TPN-POD4/1**

| Mass-charge ratio (m/z) | Retention Time (min) | Name | Molecular weight | VIP | T-test | Fold change |
| --- | --- | --- | --- | --- | --- | --- |
| 476.2782 | 7.899 | LysoPE(0:0/18:2) | 477.2855 | 1.99598 | 0.001845 | 0.502557 |
| 448.3061 | 6.228 | Chenodeoxycholic acid glycine conjugate | 449.3141 | 1.60268 | 0.014312 | 1.250801 |
| 558.3326 | 9.647 | LysoPC(18:0) | 523.3638 | 1.31459 | 0.047048 | 0.203658 |
| 431.3096 | 6.896 | Vitamin D2 | 396.3392 | 1.43322 | 0.02965 | 0.512276 |
| 464.3002 | 5.364 | Glycocholic Acid | 465.309 | 1.89095 | 0.003366 | 2.686834 |
| 243.0613 | 0.929 | Uridine | 244.0695 | 1.74885 | 0.007114 | 0.25348 |
| 164.0703 | 1.826 | L-Phenylalanine | 165.079 | 1.68424 | 0.009772 | 0.323832 |
| 179.0544 | 0.849 | myo-Inositol | 180.0634 | 1.47091 | 0.025399 | 0.2216 |
| 159.0647 | 3.110 | Pimelic acid | 160.0736 | 1.3947 | 0.034591 | 0.162482 |
| 278.0514 | 1.819 | Cytidine | 243.0855 | 1.59728 | 0.014668 | 0.232111 |
| 129.0540 | 3.133 | Ketoleucine | 130.063 | 2.50256 | 5.12E-05 | 0.673623 |
| 327.2052 | 9.733 | Androstanediol | 292.2402 | 1.95363 | 0.002363 | 0.633443 |
| 116.0699 | 1.067 | L-Valine | 117.079 | 1.97039 | 0.002145 | 0.506363 |
| 115.0382 | 1.788 | α-ketoisovaleric acid | 116.0473 | 1.8602 | 0.003981 | 0.329642 |
| 259.0271 | 3.117 | D-Glucose 6-phosphate | 260.0297 | 2.54827 | 3.46E-05 | 0.695476 |
| 263.1017 | 3.105 | Thiamine | 264.1045 | 1.95997 | 0.002278 | 1.547585 |
| 118.0489 | 0.846 | L-Threonine | 119.0582 | 1.95727 | 0.002314 | 0.578898 |
| 353.1591 | 5.248 | Ubiquinone (Q2) | 318.1831 | 1.93471 | 0.002634 | -2.6842 |
| 180.0581 | 0.851 | 4-Guanidinobutanoic acid | 145.0851 | 1.37821 | 0.036903 | 0.209615 |
| 127.0479 | 0.867 | Hydrouracil | 128.0586 | 2.26616 | 0.000319 | -4.16368 |
| 385.1676 | 3.630 | PGE3 | 350.2093 | 1.81348 | 0.005106 | -0.89735 |
| 188.9905 | 3.387 | Gentisic acid | 154.0266 | 1.57601 | 0.016141 | 1.403502 |
| 274.9847 | 3.118 | L-Cystine | 240.0238 | 2.64359 | 1.47E-05 | 0.627043 |
| 130.0830 | 1.414 | L-Leucine | 131.0946 | 2.70405 | 8.25E-06 | 0.789875 |
| 166.0565 | 3.634 | Pyridoxal | 167.0582 | 2.38757 | 0.000129 | 1.517517 |
| 162.1124 | 0.879 | L-Carnitine | 161.1052 | 2.5106 | 2.15E-05 | -0.44099 |
| 132.1019 | 1.324 | L-Leucine | 131.0946 | 2.22233 | 0.00024 | 0.478132 |
| 118.0862 | 0.952 | L-Valine | 117.079 | 1.80075 | 0.003768 | 0.292022 |
| 488.2981 | 5.369 | Glycocholic Acid | 465.309 | 1.74362 | 0.005166 | 2.768096 |
| 125.0207 | 2.761 | Acetoacetic acid | 102.0317 | 1.59321 | 0.011202 | -0.07299 |
| 206.0808 | 3.695 | Indolelactic acid | 205.0739 | 1.54256 | 0.014284 | -0.43339 |
| 450.3208 | 6.250 | Chenodeoxycholic acid glycine conjugate | 449.3141 | 1.3268 | 0.036728 | 1.839025 |
| 251.1286 | 6.563 | Ubiquinone-1 | 250.1205 | 1.32473 | 0.037037 | 0.544196 |
| 300.2873 | 7.541 | Sphingosine | 299.2824 | 1.36436 | 0.031478 | 0.799745 |
| 155.0776 | 0.804 | Ornithine | 132.0899 | 1.82284 | 0.003324 | 0.502579 |
| 90.0558 | 0.921 | β-Alanine | 89.0477 | 1.33613 | 0.035361 | -0.30543 |
| 431.3836 | 13.242 | (+)-α-Tocopherol | 430.3811 | 1.76254 | 0.00466 | 0.618074 |
| 291.0736 | 0.892 | Inosine | 268.0808 | 1.61076 | 0.010276 | 1.682414 |
| 143.0310 | 16.467 | Purine | 120.0436 | 1.3506 | 0.033323 | -0.04134 |
| 175.1165 | 0.833 | L-Arginine | 174.1117 | 2.29126 | 0.000141 | 0.717855 |
| 361.1952 | 4.596 | Cortisone | 360.1937 | 1.63419 | 0.009143 | 0.462404 |
| 202.0440 | 2.759 | Hippuric acid | 179.0582 | 1.54849 | 0.013889 | -0.06668 |
| 169.0388 | 1.071 | Uric acid | 168.0283 | 1.63578 | 0.009071 | -0.32814 |
| 287.0999 | 3.107 | Thiamine | 264.1045 | 1.5162 | 0.016157 | 1.565287 |
| 119.0730 | 2.759 | β-Hydroxyisovaleric acid | 118.063 | 1.32228 | 0.037406 | -0.07035 |
| 196.0545 | 4.060 | Salicyluric acid | 195.0532 | 1.87339 | 0.002477 | -0.25402 |
| 198.0698 | 4.068 | L-Dopa | 197.0688 | 1.71522 | 0.006016 | 2.354197 |
| 141.0111 | 2.759 | Succinic acid | 118.0266 | 1.439 | 0.022881 | -0.07088 |

**TPN-POD7/1**

| Mass-charge ratio (m/z) | Retention Time (min) | Name | Molecular weight | VIP | T-test | Fold change |
| --- | --- | --- | --- | --- | --- | --- |
| 476.2782 | 7.899 | LysoPE(0:0/18:2) | 477.2855 | 1.82388 | 0.000178 | 0.666659 |
| 448.3061 | 6.228 | Chenodeoxycholic acid glycine conjugate | 449.3141 | 1.8282 | 0.000171 | 1.987325 |
| 303.2325 | 9.929 | Arachidonic Acid | 304.2402 | 1.43267 | 0.004316 | 0.522511 |
| 558.3326 | 9.647 | LysoPC(18:0) | 523.3638 | 1.0637 | 0.037915 | 0.184679 |
| 257.1752 | 6.469 | Tetradecanedioic acid | 258.1831 | 1.41017 | 0.00503 | 0.794942 |
| 243.0613 | 0.929 | Uridine | 244.0695 | 2.11817 | 7.01E-06 | 0.556604 |
| 464.3002 | 5.364 | Glycocholic Acid | 465.309 | 1.76727 | 0.000302 | 2.966955 |
| 452.2768 | 8.275 | LysoPE(0:0/16:0) | 453.2855 | 1.71907 | 0.000465 | 0.434033 |
| 407.2789 | 6.089 | Cholic acid | 408.2876 | 1.38521 | 0.005941 | 7.00663 |
| 281.2475 | 10.645 | Oleic Acid | 282.2559 | 1.34579 | 0.007672 | 0.783045 |
| 255.2320 | 10.537 | Palmitic acid | 256.2402 | 1.29233 | 0.010707 | 0.46806 |
| 285.2062 | 7.415 | Hexadecanedioic acid | 286.2144 | 1.1369 | 0.025986 | 0.62379 |
| 178.0494 | 3.099 | Hippuric acid | 179.0582 | 1.33519 | 0.008206 | 1.338266 |
| 225.1842 | 7.160 | Myristoleic acid | 226.1933 | 1.32256 | 0.008884 | 1.241719 |
| 159.0647 | 3.110 | Pimelic acid | 160.0736 | 1.41718 | 0.004797 | 0.21934 |
| 179.0544 | 0.849 | myo-Inositol | 180.0634 | 1.01307 | 0.048554 | 0.185354 |
| 146.0443 | 0.896 | L-Glutamate | 147.0532 | 1.16092 | 0.022834 | 0.413332 |
| 129.0540 | 3.133 | Ketoleucine | 130.063 | 2.39735 | 1.24E-07 | 0.931311 |
| 327.2052 | 9.733 | Androstanediol | 292.2402 | 1.51747 | 0.002359 | 0.848505 |
| 116.0699 | 1.067 | L-Valine | 117.079 | 1.63175 | 0.000973 | 0.475042 |
| 115.0382 | 1.788 | α-ketoisovaleric acid | 116.0473 | 1.67781 | 0.000664 | 0.407315 |
| 259.0271 | 3.117 | D-Glucose 6-phosphate | 260.0297 | 2.32574 | 3.9E-07 | 0.870623 |
| 263.1017 | 3.105 | Thiamine | 264.1045 | 2.23564 | 1.47E-06 | 2.421309 |
| 118.0489 | 0.846 | L-Threonine | 119.0582 | 1.59674 | 0.001288 | 0.655325 |
| 353.1591 | 5.248 | Ubiquinone (Q2) | 318.1831 | 1.66514 | 0.000739 | -3.36261 |
| 103.0382 | 1.492 | (R)-3-Hydroxybutyric acid | 104.0473 | 1.34411 | 0.007754 | 0.711563 |
| 257.0320 | 1.160 | L-Cystathionine | 222.0674 | 1.09602 | 0.032186 | 0.251078 |
| 127.0479 | 0.867 | Hydrouracil | 128.0586 | 1.16677 | 0.022116 | -1.65725 |
| 124.0041 | 0.880 | Taurine | 125.0147 | 1.13839 | 0.02578 | 0.55807 |
| 385.1676 | 3.630 | PGE3 | 350.2093 | 1.78397 | 0.000259 | -1.18363 |
| 188.9905 | 3.387 | Gentisic acid | 154.0266 | 1.53765 | 0.00203 | 1.174163 |
| 381.1726 | 10.610 | Corticosterone | 346.2144 | 1.30027 | 0.0102 | 0.626192 |
| 274.9847 | 3.118 | L-Cystine | 240.0238 | 2.55664 | 6.77E-09 | 0.801622 |
| 130.0830 | 1.414 | L-Leucine | 131.0946 | 2.56066 | 6.24E-09 | 0.975937 |
| 166.0565 | 3.634 | Pyridoxal | 167.0582 | 2.18882 | 2.8E-06 | 1.368838 |
| 145.0564 | 0.877 | L-Glutamine | 146.0691 | 1.4281 | 0.004453 | 0.337623 |
| 162.1124 | 0.879 | L-Carnitine | 161.1052 | 2.46334 | 1.78E-08 | -0.7268 |
| 132.1019 | 1.324 | L-Leucine | 131.0946 | 2.34032 | 1.63E-07 | 0.601071 |
| 118.0862 | 0.952 | L-Valine | 117.079 | 1.86017 | 9.27E-05 | 0.352288 |
| 488.2981 | 5.369 | Glycocholic Acid | 465.309 | 1.72732 | 0.00034 | 2.747457 |
| 454.2931 | 8.282 | LysoPE(0:0/16:0) | 453.2855 | 1.67228 | 0.000558 | 0.400951 |
| 472.3030 | 6.231 | Chenodeoxycholic acid glycine conjugate | 449.3141 | 1.62205 | 0.000858 | 1.937136 |
| 305.2473 | 9.924 | Arachidonic Acid | 304.2402 | 1.17804 | 0.018866 | 0.461086 |
| 468.3084 | 7.476 | LysoPC(14:0) | 467.3012 | 1.12618 | 0.025183 | 0.310999 |
| 532.3377 | 9.014 | LysoPE(0:0/20:0) | 509.3481 | 1.03717 | 0.040116 | 0.257617 |
| 206.0808 | 3.695 | Indolelactic acid | 205.0739 | 1.84694 | 0.000106 | -0.7315 |
| 482.3236 | 7.968 | LysoPC(15:0) | 481.3168 | 1.31115 | 0.008438 | 0.349815 |
| 302.3049 | 7.753 | Sphinganine | 301.2981 | 1.24175 | 0.012988 | 0.99285 |
| 307.2624 | 10.275 | Stearic acid | 284.2715 | 1.0041 | 0.047248 | 0.532341 |
| 355.2630 | 6.093 | Adrenic Acid | 332.2715 | 1.28986 | 0.009659 | 5.448902 |
| 139.0012 | 16.465 | Fumaric acid | 116.011 | 1.11477 | 0.026788 | -0.04462 |
| 300.2873 | 7.541 | Sphingosine | 299.2824 | 1.41427 | 0.004229 | 1.308956 |
| 431.3836 | 13.242 | (+)-α-Tocopherol | 430.3811 | 1.94417 | 3.75E-05 | 0.816727 |
| 164.0106 | 16.382 | O-Phosphorylethanolamine | 141.0191 | 1.02879 | 0.041833 | -0.03912 |
| 175.1165 | 0.833 | L-Arginine | 174.1117 | 2.25915 | 6E-07 | 0.848244 |
| 361.1952 | 4.596 | Cortisone | 360.1937 | 1.24308 | 0.012883 | 0.376991 |
| 215.0204 | 1.073 | Citric acid | 192.027 | 1.06671 | 0.034513 | 0.310795 |
| 287.0999 | 3.107 | Thiamine | 264.1045 | 1.87618 | 7.84E-05 | 2.373248 |
| 348.0802 | 0.816 | Adenosine monophosphate | 347.0631 | 1.17724 | 0.018952 | 0.394479 |
| 196.0545 | 4.060 | Salicyluric acid | 195.0532 | 1.44091 | 0.003501 | -0.23248 |

**GIK-POD4/1**

| Mass-charge ratio (m/z) | Retention Time (min) | Name | Molecular weight | VIP | T-test | Fold change |
| --- | --- | --- | --- | --- | --- | --- |
| 476.2782 | 7.899 | LysoPE(0:0/18:2) | 477.2855 | 1.34802 | 0.042001 | 0.605517 |
| 303.2325 | 9.929 | Arachidonic Acid | 304.2402 | 1.68232 | 0.009531 | 0.567673 |
| 279.2323 | 10.071 | Linoleic acid | 280.2402 | 1.75272 | 0.006622 | 0.806959 |
| 431.3096 | 6.896 | Vitamin D2 | 396.3392 | 1.48613 | 0.023824 | 0.751477 |
| 243.0613 | 0.929 | Uridine | 244.0695 | 1.8359 | 0.004193 | 0.358327 |
| 407.2789 | 6.089 | Cholic acid | 408.2876 | 1.62983 | 0.012347 | 4.669571 |
| 255.2320 | 10.537 | Palmitic acid | 256.2402 | 1.60296 | 0.014039 | 0.58079 |
| 281.2475 | 10.645 | Oleic Acid | 282.2559 | 1.51832 | 0.020691 | 0.788935 |
| 464.3002 | 5.364 | Glycocholic Acid | 465.309 | 1.44978 | 0.027821 | 3.155174 |
| 285.2062 | 7.415 | Hexadecanedioic acid | 286.2144 | 1.36013 | 0.040059 | 0.904403 |
| 375.1332 | 10.486 | Riboflavin | 376.1383 | 1.63945 | 0.011783 | 0.849918 |
| 187.0962 | 3.941 | Nonanedioic acid | 188.1049 | 1.46993 | 0.025543 | -0.30516 |
| 164.0703 | 1.826 | L-Phenylalanine | 165.079 | 1.65807 | 0.010756 | 0.321042 |
| 146.0443 | 0.896 | L-Glutamate | 147.0532 | 1.45496 | 0.02722 | 0.290464 |
| 278.0514 | 1.819 | Cytidine | 243.0855 | 1.60581 | 0.013851 | 0.292113 |
| 327.2052 | 9.733 | Androstanediol | 292.2402 | 1.69173 | 0.009088 | 0.538388 |
| 129.0540 | 3.133 | Ketoleucine | 130.063 | 1.60546 | 0.013874 | 0.526248 |
| 116.0699 | 1.067 | L-Valine | 117.079 | 2.01987 | 0.001361 | 0.523448 |
| 118.0489 | 0.846 | L-Threonine | 119.0582 | 2.09225 | 0.000832 | 0.920512 |
| 259.0271 | 3.117 | D-Glucose 6-phosphate | 260.0297 | 1.59055 | 0.014885 | 0.397912 |
| 353.1591 | 5.248 | Ubiquinone (Q2) | 318.1831 | 1.77684 | 0.005818 | -2.61159 |
| 103.0382 | 1.492 | (R)-3-Hydroxybutyric acid | 104.0473 | 1.45764 | 0.026914 | 0.7826 |
| 127.0479 | 0.867 | Hydrouracil | 128.0586 | 1.80028 | 0.005118 | -4.08116 |
| 381.1726 | 10.610 | Corticosterone | 346.2144 | 1.76181 | 0.006308 | 0.769737 |
| 274.9847 | 3.118 | L-Cystine | 240.0238 | 1.63696 | 0.011927 | 0.441371 |
| 130.0830 | 1.414 | L-Leucine | 131.0946 | 2.646 | 5.39E-06 | 0.895476 |
| 166.0565 | 3.634 | Pyridoxal | 167.0582 | 2.00091 | 0.00154 | 1.268836 |
| 145.0564 | 0.877 | L-Glutamine | 146.0691 | 1.40595 | 0.033354 | 0.241128 |
| 162.1124 | 0.879 | L-Carnitine | 161.1052 | 2.40294 | 2.04E-08 | -0.79985 |
| 132.1019 | 1.324 | L-Leucine | 131.0946 | 2.18629 | 1.55E-06 | 0.804606 |
| 118.0862 | 0.952 | L-Valine | 117.079 | 2.00121 | 2.34E-05 | 0.440952 |
| 104.1069 | 0.853 | Choline | 103.0997 | 1.73227 | 0.000456 | 0.576368 |
| 165.0547 | 1.150 | m-Coumaric acid | 164.0473 | 1.71138 | 0.000554 | 0.443007 |
| 182.0811 | 1.151 | L-Tyrosine | 181.0739 | 1.69748 | 0.000629 | 0.426917 |
| 149.0597 | 1.815 | Cinnamic acid | 148.0524 | 1.63486 | 0.001091 | 0.354678 |
| 166.0862 | 1.815 | L-Phenylalanine | 165.079 | 1.61285 | 0.001312 | 0.347545 |
| 305.2473 | 9.924 | Arachidonic Acid | 304.2402 | 1.40797 | 0.0061 | 0.621713 |
| 137.0458 | 1.128 | Hypoxanthine | 136.0385 | 1.38408 | 0.007155 | -0.58322 |
| 380.2557 | 7.357 | Sphingosine 1-phosphate | 379.2488 | 1.23033 | 0.01836 | 0.393777 |
| 496.3395 | 8.483 | LysoPC(16:0) | 495.3325 | 1.22532 | 0.018888 | 0.227611 |
| 488.2981 | 5.369 | Glycocholic Acid | 465.309 | 1.10633 | 0.035678 | 3.098218 |
| 302.3049 | 7.753 | Sphinganine | 301.2981 | 1.40315 | 0.006301 | 0.906979 |
| 281.2472 | 10.067 | Linoleic acid | 280.2402 | 1.34937 | 0.008962 | 0.648407 |
| 125.0207 | 2.761 | Acetoacetic acid | 102.0317 | 1.08607 | 0.039478 | -0.06577 |
| 136.0612 | 1.069 | Adenine | 135.0545 | 1.46947 | 0.003974 | 0.307946 |
| 307.2624 | 10.275 | Stearic acid | 284.2715 | 1.43554 | 0.00505 | 0.742541 |
| 355.2630 | 6.093 | Adrenic Acid | 332.2715 | 1.20163 | 0.021564 | 3.713869 |
| 139.0012 | 16.465 | Fumaric acid | 116.011 | 1.58926 | 0.001593 | -0.10068 |
| 379.2426 | 10.112 | PGF1α | 356.2563 | 1.58139 | 0.001697 | -0.42267 |
| 300.2873 | 7.541 | Sphingosine | 299.2824 | 1.72294 | 0.000498 | 1.456656 |
| 90.0558 | 0.921 | β-Alanine | 89.0477 | 1.25324 | 0.016095 | -0.37705 |
| 150.0569 | 1.145 | L-Methionine | 149.051 | 1.06746 | 0.04325 | 0.473331 |
| 155.0413 | 2.759 | L-Asparagine | 132.0535 | 1.06148 | 0.04452 | -0.06456 |
| 183.0844 | 1.152 | D-Mannitol | 182.079 | 1.69756 | 0.000629 | 0.414673 |
| 431.3836 | 13.242 | (+)-α-Tocopherol | 430.3811 | 1.499 | 0.003205 | 0.838734 |
| 249.1853 | 8.024 | Myristoleic acid | 226.1933 | 1.2022 | 0.021497 | -0.11338 |
| 143.0310 | 16.467 | Purine | 120.0436 | 1.51605 | 0.002822 | -0.09784 |
| 164.0106 | 16.382 | O-Phosphorylethanolamine | 141.0191 | 1.40391 | 0.006269 | -0.09671 |
| 175.1165 | 0.833 | L-Arginine | 174.1117 | 1.73099 | 0.000461 | 1.025653 |
| 425.3352 | 8.051 | α-Tocotrienol | 424.3341 | 1.36021 | 0.00836 | 0.673279 |
| 361.1952 | 4.596 | Cortisone | 360.1937 | 1.42794 | 0.005323 | 0.63367 |
| 202.0440 | 2.759 | Hippuric acid | 179.0582 | 1.21472 | 0.02005 | -0.06768 |
| 169.0388 | 1.071 | Uric acid | 168.0283 | 1.31946 | 0.010817 | -0.35183 |
| 255.2260 | 9.882 | cis-9-palmitoleic acid | 254.2246 | 1.19163 | 0.022785 | 0.356852 |
| 119.0730 | 2.759 | β-Hydroxyisovaleric acid | 118.063 | 1.05166 | 0.046673 | -0.06858 |
| 196.0545 | 4.060 | Salicyluric acid | 195.0532 | 1.05618 | 0.045673 | -0.1367 |
| 198.0698 | 4.068 | L-Dopa | 197.0688 | 1.2113 | 0.020437 | 1.506884 |
| 141.0111 | 2.759 | Succinic acid | 118.0266 | 1.25327 | 0.016092 | -0.08081 |

**GIK-POD7/1**

| Mass-charge ratio (m/z) | Retention Time (min) | Name | Molecular weight | VIP | T-test | Fold change |
| --- | --- | --- | --- | --- | --- | --- |
| 162.1124 | 0.879 | L-Carnitine | 161.1052 | 2.23065 | 5.07E-09 | -1.03782 |
| 132.1019 | 1.324 | L-Leucine | 131.0946 | 1.97346 | 2.48E-06 | 0.731038 |
| 118.0862 | 0.952 | L-Valine | 117.079 | 1.64107 | 0.000282 | 0.42833 |
| 468.3084 | 7.476 | LysoPC(14:0) | 467.3012 | 1.55799 | 0.000677 | 0.760507 |
| 496.3395 | 8.483 | LysoPC(16:0) | 495.3325 | 1.52918 | 0.000899 | 0.322686 |
| 454.2931 | 8.282 | LysoPE(0:0/16:0) | 453.2855 | 1.42124 | 0.002388 | 0.624974 |
| 104.1069 | 0.853 | Choline | 103.0997 | 1.36052 | 0.003927 | 0.459565 |
| 137.0458 | 1.128 | Hypoxanthine | 136.0385 | 1.32399 | 0.005211 | -0.60318 |
| 225.1096 | 8.219 | Sebacic acid | 202.1205 | 1.2253 | 0.010591 | -0.1552 |
| 305.2473 | 9.924 | Arachidonic Acid | 304.2402 | 1.21158 | 0.011619 | 0.414362 |
| 149.0597 | 1.815 | Cinnamic acid | 148.0524 | 1.12459 | 0.020258 | 0.24888 |
| 532.3377 | 9.014 | LysoPE(0:0/20:0) | 509.3481 | 1.0164 | 0.037741 | 0.333324 |
| 482.3236 | 7.968 | LysoPC(15:0) | 481.3168 | 1.45122 | 0.001843 | 0.659015 |
| 281.2472 | 10.067 | Linoleic acid | 280.2402 | 1.22993 | 0.010262 | 0.49115 |
| 114.0665 | 0.888 | Creatinine | 113.0589 | 1.15009 | 0.017305 | 0.368069 |
| 136.0612 | 1.069 | Adenine | 135.0545 | 1.02 | 0.037009 | 0.192855 |
| 307.2624 | 10.275 | Stearic acid | 284.2715 | 1.1658 | 0.01567 | 0.542346 |
| 355.2630 | 6.093 | Adrenic Acid | 332.2715 | 1.08649 | 0.025431 | 5.757196 |
| 139.0012 | 16.465 | Fumaric acid | 116.011 | 1.51706 | 0.00101 | -0.11138 |
| 379.2426 | 10.112 | PGF1α | 356.2563 | 1.09545 | 0.024126 | -0.31082 |
| 322.2687 | 8.202 | Sphingosine | 299.2824 | 1.34687 | 0.004371 | -0.29752 |
| 90.0558 | 0.921 | β-Alanine | 89.0477 | 1.01339 | 0.038361 | -0.39266 |
| 431.3836 | 13.242 | (+)-α-Tocopherol | 430.3811 | 1.07235 | 0.027605 | 0.651269 |
| 143.0310 | 16.467 | Purine | 120.0436 | 1.45099 | 0.001847 | -0.09933 |
| 164.0106 | 16.382 | O-Phosphorylethanolamine | 141.0191 | 1.58295 | 0.000526 | -0.13139 |
| 175.1165 | 0.833 | L-Arginine | 174.1117 | 1.94795 | 3.92E-06 | 0.787767 |
| 425.3352 | 8.051 | α-Tocotrienol | 424.3341 | 1.16398 | 0.015853 | 0.479992 |
| 202.0440 | 2.759 | Hippuric acid | 179.0582 | 1.02134 | 0.036741 | -0.05044 |
| 287.0999 | 3.107 | Thiamine | 264.1045 | 1.21302 | 0.011508 | 3.234063 |
| 154.0627 | 0.892 | Creatine | 131.0695 | 1.08595 | 0.025511 | -0.71265 |
| 198.0698 | 4.068 | L-Dopa | 197.0688 | 1.30482 | 0.006018 | 1.623446 |
| 476.2782 | 7.899 | LysoPE(0:0/18:2) | 477.2855 | 2.04457 | 0.000593 | 1.046836 |
| 530.3015 | 8.469 | LysoPC(16:0) | 495.3325 | 1.30472 | 0.040637 | 0.119477 |
| 558.3326 | 9.647 | LysoPC(18:0) | 523.3638 | 1.68711 | 0.006399 | 0.290663 |
| 303.2325 | 9.929 | Arachidonic Acid | 304.2402 | 1.59267 | 0.01069 | 0.447425 |
| 279.2323 | 10.071 | Linoleic acid | 280.2402 | 1.63135 | 0.008706 | 0.625809 |
| 283.2636 | 9.632 | Stearic acid | 284.2715 | 1.61625 | 0.00944 | 0.319689 |
| 243.0613 | 0.929 | Uridine | 244.0695 | 2.34164 | 3.95E-05 | 0.568505 |
| 508.3392 | 9.649 | LysoPE(0:0/20:0) | 509.3481 | 1.76693 | 0.00401 | 0.319375 |
| 452.2768 | 8.275 | LysoPE(0:0/16:0) | 453.2855 | 1.70375 | 0.00582 | 0.569339 |
| 255.2320 | 10.537 | Palmitic acid | 256.2402 | 1.63535 | 0.008521 | 0.492741 |
| 480.3081 | 8.473 | LysoPC(15:0) | 481.3168 | 1.5776 | 0.011558 | 0.168133 |
| 407.2789 | 6.089 | Cholic acid | 408.2876 | 1.41977 | 0.024756 | 6.887157 |
| 281.2475 | 10.645 | Oleic Acid | 282.2559 | 1.35072 | 0.033521 | 0.582335 |
| 375.1332 | 10.486 | Riboflavin | 376.1383 | 1.4823 | 0.018528 | 0.619086 |
| 146.0443 | 0.896 | L-Glutamate | 147.0532 | 1.5558 | 0.012918 | 0.383371 |
| 129.0540 | 3.133 | Ketoleucine | 130.063 | 1.68569 | 0.006451 | 0.518443 |
| 327.2052 | 9.733 | Androstanediol | 292.2402 | 1.4788 | 0.018838 | 0.424089 |
| 116.0699 | 1.067 | L-Valine | 117.079 | 1.3512 | 0.033453 | 0.346982 |
| 118.0489 | 0.846 | L-Threonine | 119.0582 | 2.13012 | 0.000295 | 0.861845 |
| 263.1017 | 3.105 | Thiamine | 264.1045 | 1.77405 | 0.00384 | 2.768759 |
| 259.0271 | 3.117 | D-Glucose 6-phosphate | 260.0297 | 1.5369 | 0.014204 | 0.369987 |
| 353.1591 | 5.248 | Ubiquinone (Q2) | 318.1831 | 1.6864 | 0.006425 | -3.44915 |
| 231.0231 | 3.366 | Gluconic acid | 196.0583 | 1.61617 | 0.009444 | 1.051261 |
| 127.0479 | 0.867 | Hydrouracil | 128.0586 | 1.58721 | 0.010997 | -4.07233 |
| 188.9905 | 3.387 | Gentisic acid | 154.0266 | 1.6611 | 0.007401 | 1.635082 |
| 381.1726 | 10.610 | Corticosterone | 346.2144 | 1.34444 | 0.034429 | 0.502347 |
| 274.9847 | 3.118 | L-Cystine | 240.0238 | 1.78971 | 0.003488 | 0.491869 |
| 130.0830 | 1.414 | L-Leucine | 131.0946 | 2.21234 | 0.000142 | 0.845125 |
| 166.0565 | 3.634 | Pyridoxal | 167.0582 | 2.18489 | 0.000183 | 1.434929 |
| 145.0564 | 0.877 | L-Glutamine | 146.0691 | 1.73207 | 0.004938 | 0.299979 |

**TPN/GIK-POD4**

| Mass-charge ratio (m/z) | Retention Time (min) | Name | Molecular weight | VIP | T-test | Fold change |
| --- | --- | --- | --- | --- | --- | --- |
| 530.3015 | 8.469 | LysoPC(16:0) | 495.3325 | 1.87952 | 0.000525577 | 0.273176 |
| 303.2325 | 9.929 | Arachidonic Acid | 304.2402 | 1.73217 | 0.001642841 | -0.58031 |
| 558.3326 | 9.647 | LysoPC(18:0) | 523.3638 | 1.40843 | 0.012675136 | 0.286962 |
| 283.2636 | 9.632 | Stearic acid | 284.2715 | 2.11363 | 6.04481E-05 | 1.420618 |
| 279.2323 | 10.071 | Linoleic acid | 280.2402 | 1.75785 | 0.001361297 | -0.79872 |
| 257.1752 | 6.469 | Tetradecanedioic acid | 258.1831 | 1.37897 | 0.014865184 | -0.77491 |
| 480.3081 | 8.473 | LysoPC(15:0) | 481.3168 | 1.85896 | 0.000621913 | 0.581477 |
| 508.3392 | 9.649 | LysoPE(0:0/20:0) | 509.3481 | 1.77226 | 0.001222694 | 0.605136 |
| 281.2475 | 10.645 | Oleic Acid | 282.2559 | 1.63057 | 0.0033201 | -0.92542 |
| 255.2320 | 10.537 | Palmitic acid | 256.2402 | 1.31355 | 0.020884113 | -0.46192 |
| 375.1332 | 10.486 | Riboflavin | 376.1383 | 1.55609 | 0.005354047 | -0.83638 |
| 133.0154 | 2.752 | Malic acid | 134.0215 | 1.61678 | 0.003635828 | -0.19132 |
| 225.1842 | 7.160 | Myristoleic acid | 226.1933 | 1.33944 | 0.018296029 | -0.7655 |
| 164.0703 | 1.826 | L-Phenylalanine | 165.079 | 1.19208 | 0.037422617 | -0.24888 |
| 159.0647 | 3.110 | Pimelic acid | 160.0736 | 1.54588 | 0.005702756 | -0.2577 |
| 129.0540 | 3.133 | Ketoleucine | 130.063 | 1.28817 | 0.023710449 | -0.39123 |
| 115.0382 | 1.788 | α-ketoisovaleric acid | 116.0473 | 1.59828 | 0.004099715 | -0.35834 |
| 103.0382 | 1.492 | (R)-3-Hydroxybutyric acid | 104.0473 | 1.91834 | 0.000379211 | -1.01554 |
| 257.0320 | 1.160 | L-Cystathionine | 222.0674 | 1.36127 | 0.016328253 | -0.32425 |
| 174.0487 | 0.910 | Guanidinosuccinic Acid | 175.0593 | 1.85538 | 0.000640147 | -0.86791 |
| 197.0806 | 3.786 | L-Nicotine | 162.1157 | 1.50964 | 0.007104619 | -0.96293 |
| 381.1726 | 10.610 | Corticosterone | 346.2144 | 1.75996 | 0.001340231 | -0.79196 |
| 305.2473 | 9.924 | Arachidonic Acid | 304.2402 | 1.90602 | 0.000768 | -0.65527 |
| 162.1124 | 0.879 | L-Carnitine | 161.1052 | 1.24674 | 0.035517 | 0.240487 |
| 281.2472 | 10.067 | Linoleic acid | 280.2402 | 1.39881 | 0.017383 | -0.46485 |
| 205.0969 | 2.684 | L-Tryptophan | 204.0899 | 1.32133 | 0.025288 | 0.281775 |
| 203.0522 | 0.862 | myo-Inositol | 180.0634 | 1.28554 | 0.029841 | 0.32356 |
| 307.2624 | 10.275 | Stearic acid | 284.2715 | 1.92937 | 0.000643 | -0.8227 |
| 139.0012 | 16.465 | Fumaric acid | 116.011 | 1.91345 | 0.000726 | 0.099171 |
| 379.2426 | 10.112 | PGF1α | 356.2563 | 1.36326 | 0.020705 | 0.353273 |
| 204.1213 | 0.904 | Acetylcarnitine | 203.1158 | 1.44466 | 0.01377 | -0.42854 |
| 322.2687 | 8.202 | Sphingosine | 299.2824 | 2.08639 | 0.000178 | -0.50252 |
| 155.0413 | 2.759 | L-Asparagine | 132.0535 | 1.57782 | 0.006657 | -0.11026 |
| 90.0558 | 0.921 | β-Alanine | 89.0477 | 1.55023 | 0.007788 | 0.366225 |
| 155.0776 | 0.804 | Ornithine | 132.0899 | 1.18312 | 0.046712 | 0.372016 |
| 249.1853 | 8.024 | Myristoleic acid | 226.1933 | 1.50817 | 0.00983 | 0.120022 |
| 143.0310 | 16.467 | Purine | 120.0436 | 1.2104 | 0.041605 | -0.0568 |
| 291.0736 | 0.892 | Inosine | 268.0808 | 1.1835 | 0.046636 | 1.506234 |
| 164.0106 | 16.382 | O-Phosphorylethanolamine | 141.0191 | 1.47489 | 0.011753 | 0.063425 |
| 119.0730 | 2.759 | β-Hydroxyisovaleric acid | 118.063 | 1.50239 | 0.010143 | -0.10696 |
| 335.3033 | 10.491 | Docosatrienoic Acid | 334.2872 | 1.27115 | 0.031851 | 0.589766 |
| 196.0545 | 4.060 | Salicyluric acid | 195.0532 | 1.7006 | 0.00317 | -0.17426 |
| 341.3524 | 11.578 | Docosanoic acid | 340.3341 | 1.33579 | 0.023622 | 0.322915 |
| 141.0111 | 2.759 | Succinic acid | 118.0266 | 1.83296 | 0.001306 | -0.12521 |
| 118.0656 | 2.754 | Guanidineacetic acid | 117.0538 | 1.4341 | 0.01454 | -0.09651 |

**TPN/GIK-POD7**

| Mass-charge ratio (m/z) | Retention Time (min) | Name | Molecular weight | VIP | T-test | Fold change |
| --- | --- | --- | --- | --- | --- | --- |
| 472.3030 | 6.231 | Chenodeoxycholic acid glycine conjugate | 449.3141 | 1.13687 | 0.049798 | 1.14743 |
| 125.0207 | 2.761 | Acetoacetic acid | 102.0317 | 1.67101 | 0.002664 | -0.08982 |
| 114.0665 | 0.888 | Creatinine | 113.0589 | 1.9588 | 0.000275 | -0.48198 |
| 139.0012 | 16.465 | Fumaric acid | 116.011 | 1.69634 | 0.002233 | 0.085373 |
| 322.2687 | 8.202 | Sphingosine | 299.2824 | 2.40075 | 1.66E-06 | -0.50914 |
| 155.0413 | 2.759 | L-Asparagine | 132.0535 | 2.13244 | 4.95E-05 | -0.13379 |
| 90.0558 | 0.921 | β-Alanine | 89.0477 | 1.68469 | 0.002423 | 0.531224 |
| 150.0569 | 1.145 | L-Methionine | 149.051 | 1.46373 | 0.009787 | 0.453855 |
| 183.0844 | 1.152 | D-Mannitol | 182.079 | 1.24178 | 0.03104 | 0.208512 |
| 143.0310 | 16.467 | Purine | 120.0436 | 1.3185 | 0.021354 | -0.05912 |
| 164.0106 | 16.382 | O-Phosphorylethanolamine | 141.0191 | 1.56674 | 0.005282 | 0.073576 |
| 119.0730 | 2.759 | β-Hydroxyisovaleric acid | 118.063 | 2.25855 | 1.15E-05 | -0.13304 |
| 154.0627 | 0.892 | Creatine | 131.0695 | 1.30431 | 0.022928 | 0.697572 |
| 196.0545 | 4.060 | Salicyluric acid | 195.0532 | 2.36731 | 2.7E-06 | -0.22765 |
| 141.0111 | 2.759 | Succinic acid | 118.0266 | 2.45733 | 6.94E-07 | -0.15484 |
| 118.0656 | 2.754 | Guanidineacetic acid | 117.0538 | 1.87796 | 0.000555 | -0.11978 |
| 530.3015 | 8.469 | LysoPC(16:0) | 495.3325 | 1.76587 | 0.002112 | 0.18684 |
| 448.3061 | 6.228 | Chenodeoxycholic acid glycine conjugate | 449.3141 | 1.18121 | 0.04881 | 0.960985 |
| 283.2636 | 9.632 | Stearic acid | 284.2715 | 2.3234 | 1.48E-05 | 1.317287 |
| 433.2349 | 8.712 | LPA(0:0/18:2) | 434.2433 | 1.47929 | 0.01192 | 0.401867 |
| 508.3392 | 9.649 | LysoPE(0:0/20:0) | 509.3481 | 1.95786 | 0.000508 | 0.49216 |
| 480.3081 | 8.473 | LysoPC(15:0) | 481.3168 | 1.91017 | 0.000741 | 0.502724 |
| 164.0703 | 1.826 | L-Phenylalanine | 165.079 | 1.54304 | 0.008403 | -0.28513 |
| 133.0154 | 2.752 | Malic acid | 134.0215 | 1.41305 | 0.016817 | -0.16651 |
| 159.0647 | 3.110 | Pimelic acid | 160.0736 | 1.73477 | 0.002603 | -0.24694 |
| 249.1106 | 6.022 | Ubiquinone-1 | 250.1205 | 2.0095 | 0.000332 | 0.283659 |
| 409.2423 | 9.324 | LPA(0:0/16:0) | 410.2433 | 1.43114 | 0.015336 | 0.430376 |
| 257.0320 | 1.160 | L-Cystathionine | 222.0674 | 1.54046 | 0.008526 | -0.27964 |
| 199.0219 | 0.880 | m-Coumaric acid | 164.0473 | 1.33155 | 0.025042 | -0.48867 |
| 200.0283 | 2.761 | 7-Methylguanine | 165.0651 | 1.25252 | 0.035938 | -0.11283 |
| 166.0565 | 3.634 | Pyridoxal | 167.0582 | 1.1981 | 0.04547 | -0.57355 |
